# Penetrating the Blood-Brain Barrier with New Peptide–Porphyrin Conjugates Having anti-HIV Activity

**DOI:** 10.1021/acs.bioconjchem.1c00123

**Published:** 2021-05-25

**Authors:** Diogo A. Mendonça, Mariët Bakker, Christine Cruz-Oliveira, Vera Neves, Maria Angeles Jiménez, Sira Defaus, Marco Cavaco, Ana Salomé Veiga, Iris Cadima-Couto, Miguel A. R. B. Castanho, David Andreu, Toni Todorovski

**Affiliations:** † Department of Experimental and Health Sciences, Pompeu Fabra University, 08003 Barcelona, Spain; ‡ Instituto de Medicina Molecular, Faculdade de Medicina, Universidade de Lisboa, 1649-028 Lisbon, Portugal; § Department of Biological Physical Chemistry, Institute of Physical Chemistry Rocasolano (IQFR-CSIC), 28006 Madrid, Spain; ∥ Avans University of Applied Sciences, 5223 DE Breda, Netherlands

## Abstract

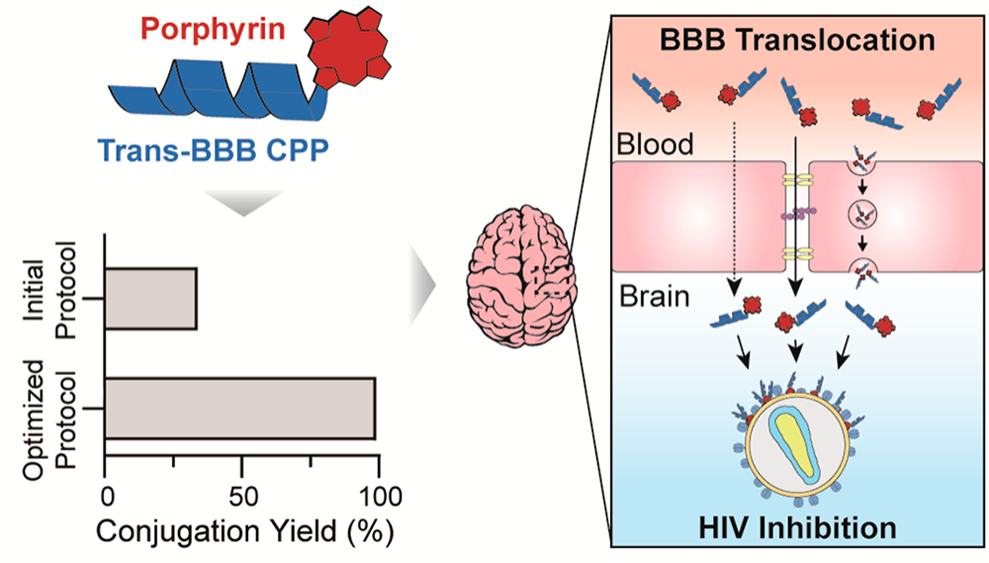

Passing through the blood-brain barrier (BBB) to treat neurological conditions is one of the main hurdles in modern medicine. Many drugs with promising in vitro profiles become ineffective in vivo due to BBB restrictive permeability. In particular, this includes drugs such as antiviral porphyrins, with the ability to fight brain-resident viruses causing diseases such as HIV-associated neurocognitive disorders (HAND). In the last two decades, BBB shuttles, particularly peptide-based ones, have shown promise in carrying various payloads across the BBB. Thus, peptide–drug conjugates (PDCs) formed by covalent attachment of a BBB peptide shuttle and an antiviral drug may become key therapeutic tools in treating neurological disorders of viral origin. In this study, we have used various approaches (guanidinium, phosphonium, and carbodiimide-based couplings) for on-resin synthesis of new peptide–porphyrin conjugates (PPCs) with BBB-crossing and potential antiviral activity. After careful fine-tuning of the synthetic chemistry, DIC/oxyma has emerged as a preferred method, by which 14 different PPCs have been made and satisfactorily characterized. The PPCs are prepared by coupling a porphyrin carboxyl group to an amino group (either *N*-terminal or a Lys side chain) of the peptide shuttle and show effective in vitro BBB translocation ability, low cytotoxicity toward mouse brain endothelial cells, and low hemolytic activity. Three of the PPCs, MP-P5, P4-MP, and P4-L-MP, effectively inhibiting HIV infectivity in vitro, stand out as most promising. Their efficacy against other brain-targeting viruses (Dengue, Zika, and SARS-CoV-2) is currently under evaluation, with preliminary results confirming that PPCs are a promising strategy to treat viral brain infections.

## Introduction

Peptide–drug conjugates (PDCs) have emerged as promising therapeutic tools for treating cancer and cardiovascular, brain, and infectious diseases, and others.^1^ By integrating two bioactive elements into a single entity, PDCs may provide novel functionalities and bioavailabilities to treat conditions where conventional drugs are ineffective.^1−5^ A case in point is brain diseases where the nonpermeability of the blood-brain barrier (BBB) severely limits therapeutic options. BBB is a natural protective structure that keeps the brain safe by specifically restricting the circulation of ions, molecules, and cells between the brain and bloodstream. Its unique structure, based on specialized endothelial cells, allows tight regulation of central nervous system (CNS) homeostasis, which is critical for proper neuronal function, as well as protection from toxins, pathogens, inflammation, and so forth. In the last two decades, considerable efforts have been devoted to strategies enabling modulation and traversing of the BBB.^6−11^ Among them, peptide-based BBB shuttles have proven particularly fruitful, with a number of sequences reported to effectively pass the BBB.^12−18^ Recently, Neves et al. showed that some domains of the Dengue virus type 2 capsid protein (DEN2C) can become effective BBB shuttles.^19^ One such sequence, after penetrating the brain by adsorptive-mediated transport (AMT), is rapidly excreted back into the bloodstream, acting as a trans-BBB vector that not only ferries drug payloads into but also flushes toxins out, preventing their brain accumulation. Thus, it would seem that, by proper choice of BBB peptide shuttles, PDCs with apposite drug cargoes can virtually be developed for a broad variety of conditions affecting the CNS.

In the complex scenario caused by the current SARS-CoV-2 pandemic, as well as by other recent virus outbreaks, it is clear that effective antiviral agents will remain a main goal in drug development programs. A special challenge in this context is that of brain-penetrating viruses such as Zika, Dengue, SARS-CoV-2, or HIV, among others, all of which pose severe health risks.^20−24^ In particular, HIV-associated neurocognitive disorders (HAND) affect approximately half of the HIV-infected population.^25^ HAND is caused by HIV entering the CNS at the early phase of infection,^26,27^ persisting in that system for decades and inducing decline in thinking or cognitive functions such as memory, reasoning, judgment, problem solving, and concentration. Studies have shown that the low BBB penetration of antiretroviral drugs was associated with continued HIV replication in the CNS and higher viral loads, precluding HAND effective treatment.^28^

Porphyrins have been recently reported as efficient against enveloped viruses,^29^ a group that includes the aforementioned brain-penetrating ones. Porphyrins are tetrapyrrole macrocycles, linked by methine bonds, with the four pyrrole nitrogens defining a metal ion coordination site involved in biological processes such as respiration and photosynthesis,^30^ as well as in diagnostic and therapeutic applications.^31,32^ Their antiviral activity can be explained by two proposed mechanisms: (i) photoactivation,^33^ a task not simple to perform when treating brain-resident viruses; (ii) viral envelope targeting, a primary mechanism in the absence of light, where virus infectivity is blocked by porphyrin local accumulation and perturbation of the viral lipid bilayer, an approach suitable to inhibiting brain-resident viruses. Unfortunately, like other antivirals, most porphyrins are unable to cross the BBB, which hinders their use against viral brain infections.^34,35^

We hypothesized that, by conjugating a porphyrin antiviral to a trans-BBB peptide shuttle, one might circumvent the challenges to fighting viral CNS diseases, in particular, HAND. Methods for peptide–porphyrin conjugation, both in solution and in solid phase, have been described,^36−38^ with a growing consensus that, in terms of simplicity and expediency, on-resin conjugation offers palpable advantages over solution procedures. However, comparative studies showing how a given strategy (e.g., solution vs on-resin, coupling chemistries, etc.) is favored over others are lacking. Herein, we report the on-resin synthesis of various new peptide–porphyrin conjugates (PPCs) with BBB-crossing ability and potential antiviral activity, and compare the effectiveness of activation methods (guanidinium, phosphonium, and carbodiimide) for coupling porphyrins to resin-bound peptides. In our study, we have used six peptide shuttles (P1–P6, Table 1) and mesoporphyrin IX (MP) and protoporphyrin IX (PP) as antiviral payloads. Two shuttles, P1 and P3, have been reported as BBB-translocating,^19^ another (P5) has been described as the “negative image” of P3,^39^ i.e., the result of switching cationic residues (Lys, Arg) in P3 to anionic (Glu), thereby changing the overall positive charge in P3 to negative in P5; other entries in Table 1 (P2, P4, P6) are versions of P1, P3, and P5, respectively, elongated with an extra Lys residue to allow *C*-terminal conjugation (Supporting Information Scheme S1). In some instances, an 8-amino-3,6-dioxaoctanoic acid (O_2_Oc) residue (abridged L, Table S1) is inserted between the peptide and the porphyrin units to ascertain if a spacer may modulate conjugate performance. In total, 14 novel conjugates have been obtained in high purity and satisfactorily characterized (Table S1, Figure S1). Three of them are effective in a BBB crossing cellular assay and display antiviral activity against HIV with low cytotoxicity.

**Table 1:**
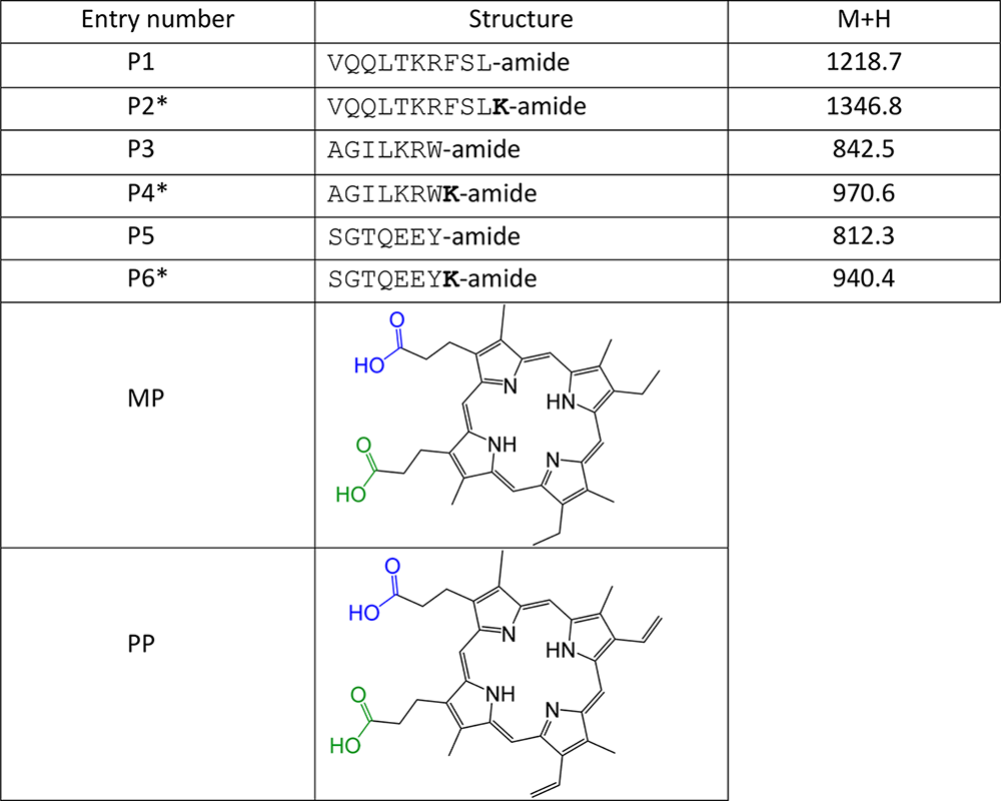
Peptide Shuttles and Porphyrins Used in This Study^a^

a P2, P4, P6 are versions of P1, P3 and P5, respectively, elongated with an extra Lys residue (in bold) to allow conjugation at the C-terminus.

## Results and Discussion

### Chemistry

**Scheme 1:**
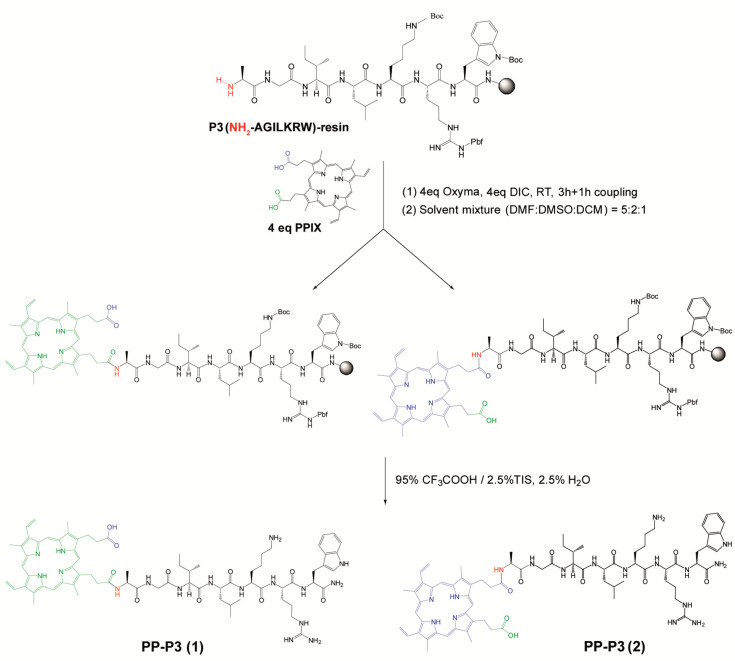
Schematic Representation of on-Resin Synthesis of Various PPCs Formation of two putative diastereomers is shown through conjugation of −COOH groups of PP with *N*-terminal NH_2_ group of P3 peptide (in Scheme S1, see more details about PP conjugation with P4 peptide through Lys side-chain NH_2_ group).

Conjugation between porphyrins and peptides has received considerable attention in the past decade due to the enhanced properties of the newly generated species, including the ability to overcome some of the obstacles related to porphyrin application.^37,38,40,41^ For example, peptide conjugation can improve poor water solubility of some porphyrins, reduce their aggregation tendency, and/or enhance porphyrin biological efficacy thanks to higher cell uptake of the conjugate vs the single porphyrin molecule.^37,42,43^ In parallel with the broader biological applications of PPCs, the need for optimized approaches allowing quantitative conjugation at various sites has increased. So far, several strategies using a variety of chemistries have been adopted, neither giving clear advantages over others.^36^ In our case, we opted for a solid-phase approach, which is advantageous for its expediency. All PPCs in this work were made by an approach (Scheme 1 and Scheme S1) where a carboxyl of MP or PP is coupled to an amino group (*N*-terminal or Lys side chain) of the CPP while it remains on the solid support, followed by acidolytic deprotection and cleavage. Thus, by combining MP and PP with six CPPs (P1 to P6) and one linker (O_2_Oc), a total of 14 PPCs were prepared (Table S1, Figure S1).

**Figure 1:**
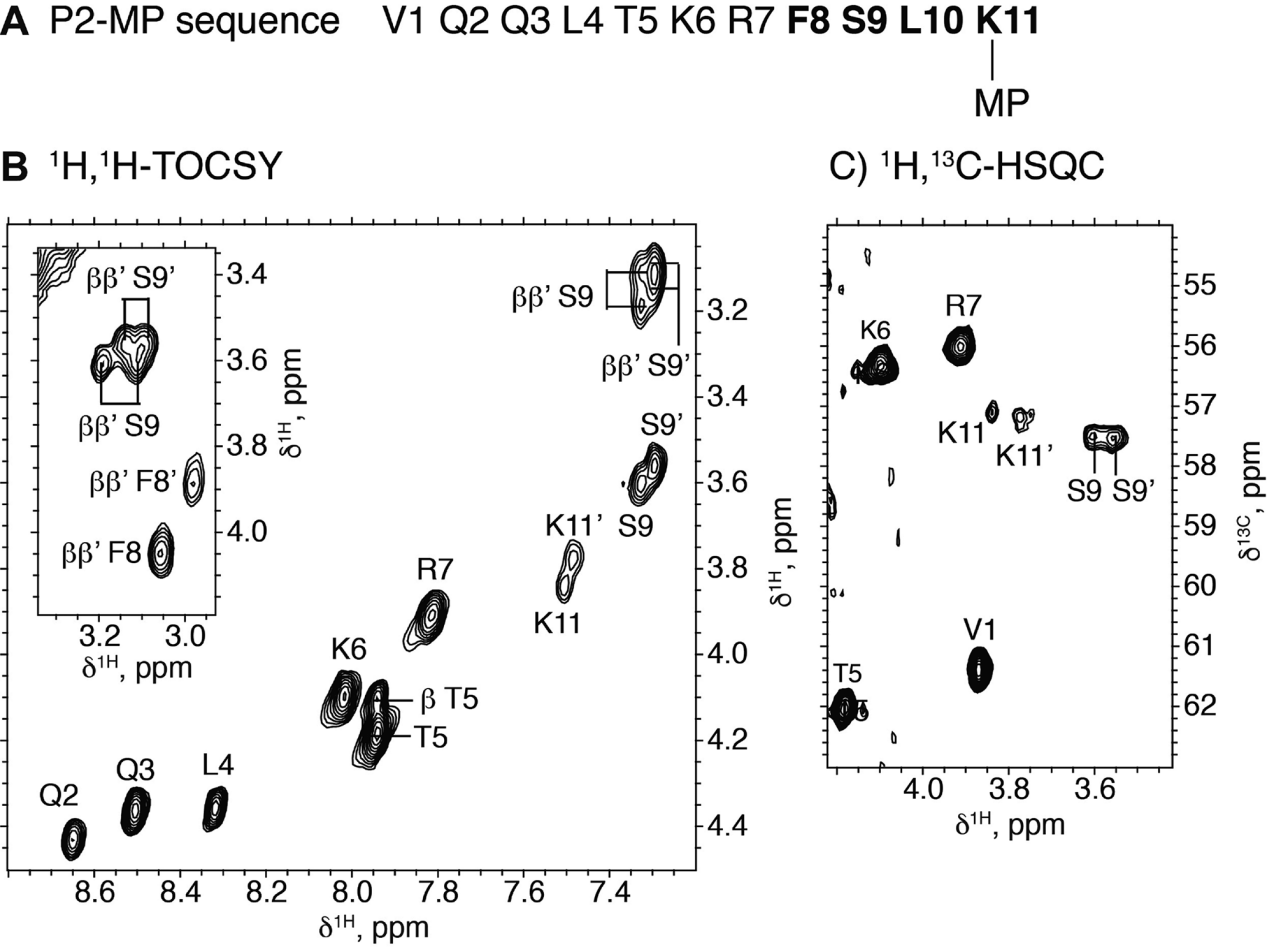
NMR data for conjugate P2-MP. (A) Peptide sequence where residues for which two sets of NMR signals are observed are highlighted in bold. (B) 2D ^1^H,^1^H-TOCSY spectral region with α-H^N^ cross-peaks labeled. Some β-H^N^ cross-peaks are also seen in this region. The inset shows α-ββ′ cross-peaks for F8 and S9 in the two diastereomers. (C) 2D ^1^H,^13^C-HSQC spectral region showing ^1^Hα-^13^Cα cross-peaks. Experimental conditions: 2 mM peptide in H_2_O/D_2_O 9:1 v/v, pH 3.1, 45 °C.

In devising this approach, we were aware that both MP and PP, selected for their activity against enveloped viruses,^29^ each have two carboxyl groups in very similar but not strictly identical environments; i.e., the carboxyl in blue in Scheme 1 (Table 1, Scheme S1) is nine carbons away from the nearest vinyl group (ethyl in MP), while the one in green is eight carbons removed, and so forth. Coupling of those slightly unsymmetric units to a homochiral peptide will result in a mixture of two diastereomers of such structural similarity to make resolution predictably difficult. Nevertheless, we deemed this route the most expeditious one, as the more orthodox course of selectively protecting each carboxyl in MP or PP appeared too arduous and of uncertain outcome. With the above caveat in mind, we actively searched for evidence of diastereomers in the end products. LC-MS inspection by carefully chosen conditions (different ion-pairing reagents, shallow gradients, etc.; see Supporting Information Figures S2 and S3) affording high resolution invariably led to observing just a single peak of the expected mass. NMR analysis, on the other hand, proved more rewarding in that two different sets of signals were detectable for Ser-Phe-Leu-Lys residues in the P2-MP conjugate, as clearly observed in the 2D ^1^H,^1^H-TOCSY and 2D ^1^H,^13^C-HSQC spectra (Figure 1). These two sets of signals can be assigned to the two putative diastereomers. That only the ^1^H and ^13^C chemical shifts of the residues closer to the MP moiety are affected (Figure 1, Table S2, Figures S4 and S5) is understandable by the very similar chemical environments in the two diastereomers. It was not possible to assign each set of signals to a specific diastereomer, because no distinctive NOE cross-peaks could be observed. In any case, the fact that the two sets of cross-peaks show approximately equal intensities indicates that the coupling reaction occurs with no regioselectivity.

Another concern of this work, of more practical consequence, was finding optimal conditions for high PPC yields by an on-resin approach. To this end, we explored three activation methods: (i) DIC/oxyma, (ii) guanidinium (TBTU or HATU), or (iii) phosphonium (PyBOP). Depending on the protocol, various reaction times were examined (Table 2). For DIC/oxyma, longer times (up to 3 h) were preferable, while couplings mediated by more reactive guanidinium or phosphonium species were completed within 1 h. Optimized conditions were first developed for *N*-terminal conjugation, then extended to side chain conjugation (Scheme S1). The influence of solid support (ChemMatrix or Protide) and porphyrin concentration (0.02 vs 0.05 M) was also assessed.

**Table 2:** Conjugation Conditions (Exploratory Runs) and Conversion Rates

porphyrin–peptide combinations	activation method/time (min)	conversion^a^ (%)
PP-P1	PyBOP-DIPEA/30	**96.8**
	HATU-DIPEA/60	**100**
	TBTU-DIPEA/30	**97.6**
	DIC-Oxyma/180 + 60	**98.7**
MP-P3	PyBOP-DIPEA/30	78.9
	HATU-DIPEA/60	59.9
	TBTU-DIPEA/30	**98.6**
	DIC-Oxyma-DIPEA/180 + 60	**99.5**
PP-P3	TBTU-DIPEA/30	90.1
	DIC-Oxyma/180 + 60	**98.5**
MP-P1	TBTU-DIPEA/30	67.3
	DIC-Oxyma-DIPEA/180 + 60	**97.1**
P2-MP	DIC-Oxyma-DIPEA/180 + 60	**99.6**
P2-PP	DIC-Oxyma/180 + 60	**99.4**

a Conversions >95% are in boldface.

**Figure 2:**
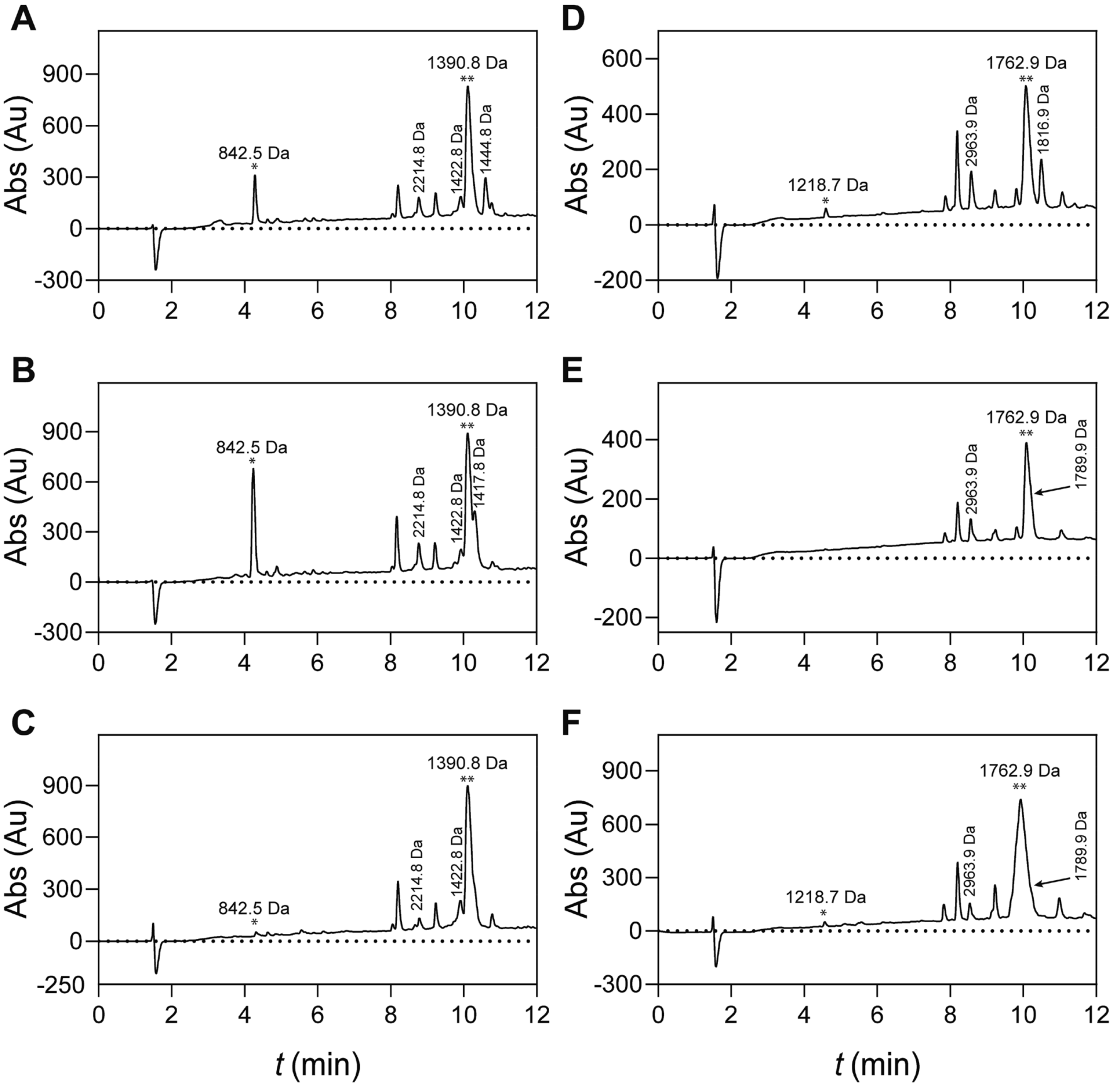
TIC of various conjugation strategies using MP-P3 (A–C) and PP-P1 (D–F) combinations. The ratio of peak area of conjugate (two asterisks) to that of starting peptide (one asterisk) is strongly influenced by peptide/porphyrin combinations for phosphonium (A and D) or guanidinium (B and E) mediated couplings, while for DIC/Oxyma mediated coupling, the peptide/porphyrin combination does not play a role in the final conversion rate (C and F). More detailed information for the other labeled side products is given in Supporting Information.

**Figure 3:**
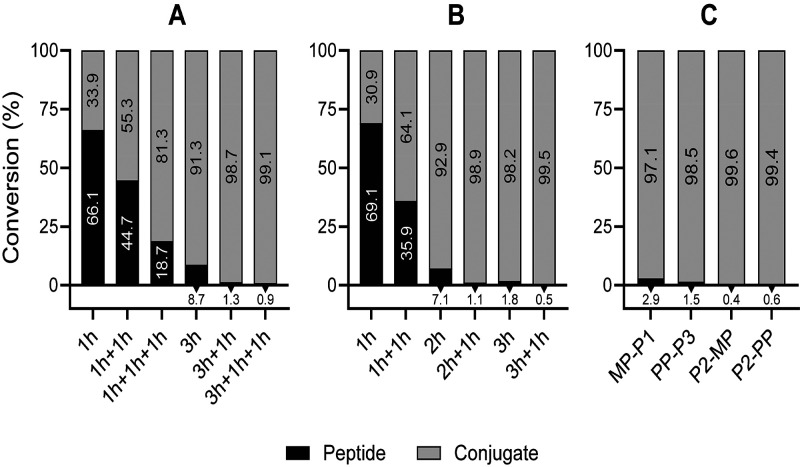
Optimization of DIC/Oxyma/(DIPEA)-mediated conditions for PP-P1 (A) and MP-P3 (B) conjugation. The best coupling protocol (3h+1h) was applied on both *N*-terminal or side-chain conjugation combinations (C). Bars refer to peptide and conjugate integrated areas in the HPLC chromatograms of the reaction crudes.

LC-MS analysis (Figure 2) of end products from the three activation protocols allowed some conclusions: (i) For guanidinium or phosphonium-mediated couplings, the conversion rate—ratio of conjugate (two asterisks) to starting peptide (one asterisk) peak areas—varies substantially across porphyrin/peptide combinations (Figure 2A,B,D,E; Table 2). (ii) For DIC/oxyma, coupling time strongly influences conversion rate (Figure 2C,F; Figure 3; Table 2). (iii) Longer times do not affect the conversion rate of guanidinium or phosphonium couplings, which on the other hand tend to generate more byproducts (details in Supporting Information, Mass Spectrometry, and Table S3). Analytical data also confirmed DIC/oxyma as the optimal activation method in terms of yield and purity. Marginal gains in conversion by guanidinium or phosphonium methods (Figure 2D,E; Table 2) tend to be offset by complex crudes whose demanding purification results in lower yields.

Having chosen DIC/oxyma as the preferred coupling method, we sought to optimize the reaction time and the number of couplings needed to achieve >95% conversion rates. As seen in Figure 3, a 3 h reaction followed by a 1 h recoupling (termed 3h+1h) led to nearly quantitative (>98%) conversion for MP-P3 and PP-P1 (Figure 3A,B) as well as for the reverse combinations (>97%, Figure 3C). The optimized 3h+1h conditions afforded almost quantitative yields too in the side-chain coupling of MP or PP to P2, thus evidencing that coupling efficiency was independent of amino group position. For MP, neutralization of the hydrochloride helped avoid sluggish couplings (Figure S6). The 3h+1h conjugation was also slowed if MP or PP concentration were lowered (from 0.05 to 0.02 M); for near-quantitative conversions, an additional coupling (3h+1h+1h) was required (data not shown).

We also checked the influence of the solid support on the conjugation results and could find no definite advantage between ChemMatrix Rink amide (100% PEG-based) and Protide (PEG with a polystyrene core) resins, which were used in Prelude (conventional batch) and Liberty Blue (microwave-assisted) synthesizers, respectively (data not shown).

All the above optimization measures were finally applied to scaled-up (50 μmol) PPC syntheses in which two additional CPPs, P5—a rather efficient BBB shuttle described as the “negative picture” of P3^39^ (Table 1)—and P6—for *C*-terminal conjugation—were used (Table 1). In this scaled-up format, the optimized protocols worked rather well, PPCs being obtained in >90% purity and ca. 30% overall (synthesis + purification) yields (Table S1).

### BBB in Vitro Translocation

**Figure 4:**
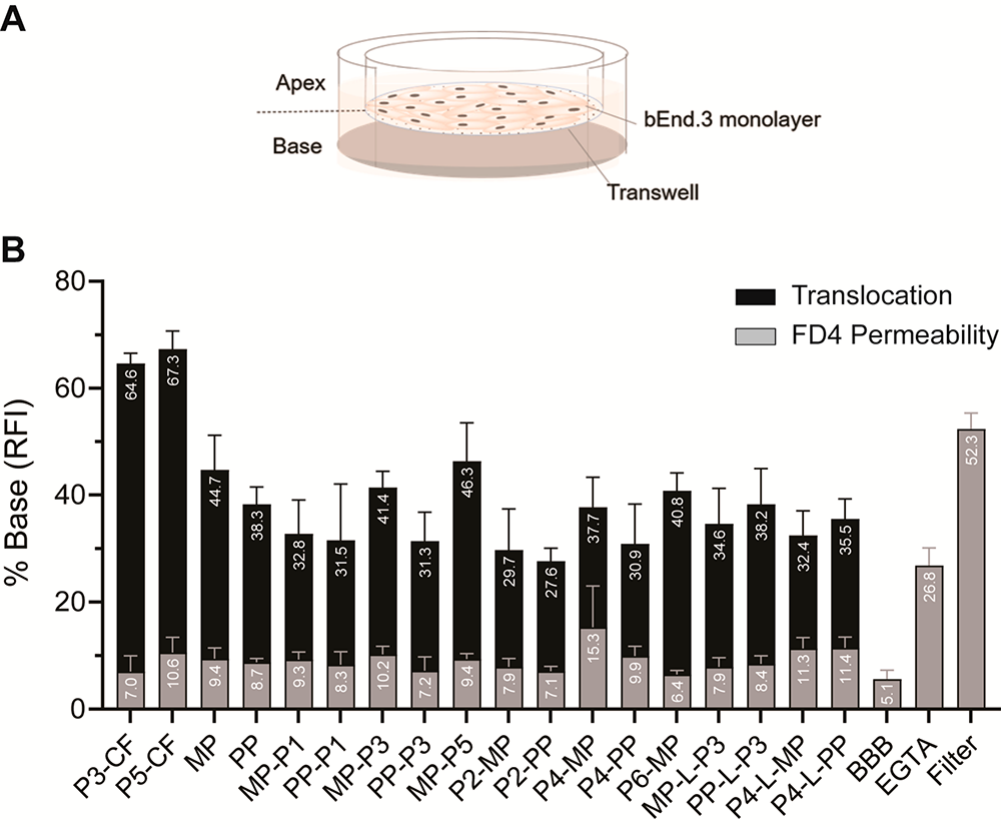
PPCs translocation across a bEnd.3 in vitro BBB model. (A) bEnd.3 in vitro BBB model transwell system. The insert, or apex, corresponds to the blood side, while the base (bottom chamber) corresponds to the brain side. (B) After formation of a monolayer, 10 μM of each conjugate was added to the apical side of the tissue culture inserts. After incubation for 24 h, samples from the basolateral side were collected, and the percentage of translocation of each conjugate (black) was calculated. FD4 permeability (gray) was measured to determine monolayer integrity post-PPCs translocation.

Assessment of PPC efficacy in BBB translocation was done in a transwell setup (Figure 4A) using bEnd.3 cell monolayers.^44^ The bEnd.3 in vitro BBB model consists of a monolayer of cells expressing a range of essential BBB transporters, also bearing the low permeability characteristic of the BBB, suitable for this study.^44−46^ All conjugates achieved significant BBB translocation, some of them reaching levels higher than 40% (Figure 4B). With the exception of P4-MP, the conjugates did not significantly disturb monolayer integrity (Figure 4B). Unexpectedly, and in contrast with in vivo,^34,35^ MP and PP translocated the monolayer. While this may be exclusive for this simple in vitro model, more studies are needed to elucidate the translocation mechanisms of porphyrins and PPCs.

### HIV Inhibition

Given the potential of PPCs for BBB translocation, we next evaluated their antiviral activity. As mentioned before, porphyrins are broad-spectrum antivirals against enveloped viruses, with HIV as an example.^29,33^ Five conjugates, MP-P3, MP-P5, P4-MP, MP-L-P3, and P4-L-MP, were shown to inhibit HIV in vitro, with IC_50_ values in the 16.12–34.03 μM range for 100 TCDI_50_ and 1.38–10.69 mM for 10 TCDI_50_ (Table 3, Figure S7). The remaining conjugates did not inhibit HIV at non-cytotoxic concentrations (data not shown). Nonconjugated porphyrins caused no significant inhibition of HIV infectivity, in agreement with results in the absence of photoactivation.^33^ Overall, it is reasonable to conclude that CPP conjugation improves porphyrin efficacy by increasing membrane affinity and/or internalization, critical for the PPCs antiviral activity,^47,48^ in agreement with the PPCs internalization results for bEnd.3 cells (Figure S8).

**Table 3:** HIV inhibition by Peptide-Porphyrin Conjugates

	IC_50_ (μM)				
Conjugates	MP-P3	MP-P5	P4-MP	MP-L-P3	P4-L-MP
**100 TCDI**_**50**_	16.12 ± 1.3	33.1 ± 1.38	34.03 ± 1.25	16.59 ± 1.29	26.35 ± 1.24
**10 TCDI**_**50**_^a^	1.38 ± 1.1	10 ± 2.9	5.77 ± 1.17	1.985 ± 1.27	10.69 ± 1.33

Control	MP		PP	
**100 TCDI**_**50**_	>50		>50	

a Approximate physiological conditions presented in a HIV brain infection.

### Cytotoxicity

All PPCs, aside from MP-P3 at 50 μM, did not alter bEnd3 cell viability, while some with *N*-terminal MP were hemolytic. Nonetheless, 9 out of 14 conjugates, including MP-P5, P4-MP, and P4-L-MP, totally lacked hemolytic activity (Table 4).

**Table 4:** Cytotoxicity of Peptide–Porphyrin Conjugates^a^

Conjugates	MP-P1	PP-P1	MP-P3	PP-P3	MP-P5
**CC**_**50**_**(μM)**	>50	>50	48.19	>50	>50
**HC**_**50**_**(μM)**	11.33	19.92	9.1	>50	>50

Conjugates	P2-MP	P2-PP	P4-MP	P4-PP	P6-MP
**CC**_**50**_**(μM)**	>50	>50	>50	>50	>50
**HC**_**50**_**(μM)**	11.31	>50	>50	>50	>50

Conjugates	MP-L-P3	PP-L-P3	P4-L-MP	P4-L-PP
**CC**_**50**_**(μM)**	>50	>50	>50	>50
**HC**_**50**_**(μM)**	8.88	>50	>50	>50^a^

Controls	MP	PP	P1	P3	P5
**CC**_**50**_**(μM)**	>50	>50	>50	>50	>50
**HC**_**50**_**(μM)**	>50	>50	>50	>50	>50

a CC_50_: 50% cytotoxic concentration toward bEnd.3 cells; HC_50_: 50% lytic concentration for human erythrocytes.

## Conclusions

In conclusion, we have developed successful on-resin synthesis of new PPCs by DIC/oxyma activation strategy as most suitable and effective approach. All conjugates were well characterized, with purity >90%, displaying a single elution peak in RP-HPLC analyses. However, due to the chemical similarity of both −COOH groups (in MP and PP), the synthesized PPCs are probably a mix of two putative diastereomers—as NMR data indicates. Three of the PPCs, MP-P5, P4-MP, and P4-L-MP, were able to effectively pass the BBB and inhibit HIV in vitro while lacking any cytotoxic or hemolytic activity. Currently, in vitro experiments of the aforementioned PPCs (including new generation PPCs) against other brain-resident viruses (Dengue, ZIKA, and SARS-CoV-2) are ongoing.

## Experimental Procedures

Mesoporphyrin IX dihydrochloride and protoporphyrin IX were from Frontier Scientific, Inc. (Logan, UT, USA). HPLC-grade DMF, DCM, and acetonitrile were obtained from Fisher (Madrid, Spain), and NMP was from Sigma-Aldrich (Madrid, Spain). Fmoc-amino acids were from Iris Biotech (Marktredwitz, Germany). Water was purified in-house with a Milli-Q Advantage A10 system (Merck, Madrid, Spain).

### Peptide Synthesis

Peptides P1–P6 (Table 1) were built on Rink amide ChemMatrix (0.5 mmoL/g) or Protide resins (0.54 mmol/g), using Fmoc chemistry, at 0.1 mmoL scale in either Prelude (Gyros, Tucson AZ) or Liberty Blue (CEM, Matthews, NC) instruments. Side chain protections were *tert*-butyl (Ser, Thr), trityl (Gln), Boc (Lys, Trp), and 2,2,4,6,7-pentamethyldihydrobenzofuran-5-sulfonyl (Arg). In P2, P4, and P6 syntheses, the extra Lys at the *C*-terminal was protected with selectively removable monomethoxytrityl (Mmt). In MP-L-P3, P4-L-MP, PP-L-P3, and P4-L-PP sequences (Table S1), the O_2_Oc spacer was coupled manually by DIC/oxyma and coupling checked by the Kaiser test.^49^ After completing each synthesis, a resin test cleavage (RTC) to check identity and purity was performed. Briefly, ca. 2 mg resin was placed in an eppendorf tube and treated with 170 μL of TFA-TIS-H_2_O (95:2.5:2.5 v/v/v) for 90 min at r.t., then 1 mL of cold diethyl ether was added to precipitate the crude peptide. After centrifugation (12 400 rpm, 8 min), supernatant was removed, and the precipitate was dried, redissolved in 15% MeCN in H_2_O, and analyzed by LC-MS (see below).

### Conjugation Chemistry

For on-resin conjugations to peptides P1–P6, solvent cocktails optimized to solubilize MP and PP were, respectively, NMP:DCM:DMF 3:2:1 and DMF:DMSO:DCM 5:2:1 (v:v:v). Trial conjugations to determine optimal chemistry were performed on 5 μmol peptide–resin with a 0.05 M final concentration of either PP or MP, in the presence of 4 equiv activator (3.9 equiv for HATU) and 8 equiv DIPEA. In DIC/oxyma-mediated MP couplings, DIPEA (8 equiv) neutralization of the hydrochloride salt was essential to avoid slow coupling (Figure S6). Various activation mixtures were tested in *N*-terminal conjugations: (i) PP to P1-resin, PyBOP/DIPEA (30 min); HATU/DIPEA (1 h); TBTU/DIPEA (30 min), or DIC/oxyma (3 + 1 h) (see Figure 2, Table 2); (ii) MP to P3-resin, PyBOP/DIPEA (30 min); HATU/DIPEA (1 h); TBTU/DIPEA (30 min) or DIC/oxyma/DIPEA (3 + 1 h); (iii) PP to P3-resin, TBTU/DIPEA (30 min) or DIC/oxyma (3 + 1 h); (iv) MP to P1-resin, TBTU/DIPEA (30 min) or DIC/oxyma/DIPEA (3 + 1 h). Figure 2 shows representative HPLC traces of these trial runs from which optimal conditions were derived for the PP (DIC/oxyma, 3h+1h) and MP (DIC/oxyma/DIPEA, 3h+1h) conjugations to the (Lys) side-chain of P2-resin, and then also generalized to other entries on Table 2. When peptide sequences with *C*-terminal Lys were used (P2, P4, P6), the side-chain Mmt group was selectively removed prior to porphyrin coupling by 1% TFA in DCM (5 × 1 min) followed by DCM washes; the cycle was repeated until no more yellow color (presence of Mmt) was observed. The resin was then neutralized for 30 s with 5% DIPEA in DCM, and porphyrin coupling was performed as above. In RTCs for these trial conjugations, cold diethyl ether (which might partially solubilize conjugates) was replaced by drying of the solution under a N_2_ stream. The residue was then taken up in 1.5 mL of 15% MeCN/H_2_O and analyzed by LC-MS (see below). Conversion rates were calculated from integrated peak areas of conjugate and starting peptide in the LC chromatograms.

Once an optimized conjugation protocol was chosen, large-scale (50 μmol) manual coupling of MP or PP to resin-bound P1–P6 was performed, followed by side chain deprotection and cleavage with 4 mL of 95% TFA, 2.5% TIPS, 2.5% H_2_O for 3 h. The cleavage solution was dried in a N_2_ stream, redissolved in 30% MeCN/H_2_O, lyophilized, and purified by semipreparative HPLC. Overall, 14 PPCs were synthesized (Table S1) in 13–28% yield range (after HPLC purification).

### Conjugate Purification

Crude lyophilized conjugates were dissolved in either 22% MeCN/H_2_O (P3 and P4) or 22% MeCN/25% DMF/H_2_O (P1, P2, P5, and P6) and purified by semipreparative HPLC on a LC20-AP instrument (Shimadzu, Kyoto, Japan) using Gemini C_18_ column (10 μm, 110 Å, 10 × 250 mm, Phenomenex) and a linear 15%–95% MeCN gradient into 0.1% TFA in H_2_O over 40 min at 6 mL/min flow rate. Fractions were analyzed by LC-MS, and those with >90% homogeneity were collected, lyophilized, and stored at −20 °C.

### LC, LC-MS, and MALDI-MS

Purified conjugates were dissolved at 1 mg/mL in 15% MeCN in 0.05%TFA/H_2_O for LC, LC-MS, and MALDI-MS characterization. For LC and LC-MS, 15 μL were injected in an LC-20AD or LCMS-2010 EV (Shimadzu, Kyoto, Japan) instruments, correspondingly, using Aeris XB-C_18_ column (particle size 3.6 μm, 150 × 4.6 mm, Phenomenex, Torrance, California) and analyzed by linear 15%–75% MeCN gradients into 0.1% TFA in H_2_O over 30 min at 1 mL/min flow rate (for LC) or linear 10%–60% MeCN gradients into 0.1% FA in H_2_O over 15 min (for LC-MS) with detection over a 200–2000 *m*/*z* mass range (Figure S1).

MALDI-MS spectra were acquired in a 4800 Proteomics Analyzer (AB Sciex, Darmstadt, Germany) operated in positive ion mode at an acceleration voltage of 20 kV, 80% grid voltage, 1.227 ns delay time, and 2.19 kV detector voltage. Equal volumes of conjugate (1 mg/mL) and matrix solution (α-cyano-4-hydroxy-cinnamic acid, 15 mg/mL in 50% MeCN in H_2_O) were mixed on the MALDI plate and air-dried. Spectra were recorded in reflector TOF mode in the 600–3000 *m*/*z* range by accumulating 30 subspectra at a fixed laser intensity of 4900.

### NMR Spectroscopy

For NMR spectra acquisition, 2 mg of lyophilized P2-MP (Tables 1 and 2) was dissolved in 0.5 mL of H_2_O/D_2_O (9:1 v/v). The pH measured with a glass microelectrode was 3.2. 1D ^1^H, 2D ^1^H,^1^H-TOCSY, 2D ^1^H,^1^H-NOESY, and 2D ^1^H–^13^C-HSQC were recorded at 5, 25, and 45 °C using a Bruker Avance-600 spectrometer equipped with a cryoprobe, as previously described.^50^ Data were processed using the TOPSPIN software (Bruker Biospin, Karlsruhe, Germany) and analyzed using the NMRFAM-SPARKY software.^51^

As reported for a nonpeptide MP conjugate,^52^ NMR signals of P2-MP were sharper at higher temperatures and were therefore assigned at 45 °C (Table S2), whereas those of the MP moiety could not be assigned because they remained broad even at 45 °C.

### Biological Assays of Peptide–Porphyrin Conjugates

10 mM conjugate solutions in DMSO were sonicated (Transsonic 460/H, Elma, Switzerland) for 10 min; a fraction of the solution at 10 mM was divided into 10 μL aliquots, and the rest was further diluted to 2 mM and also divided into aliquots. All aliquots were stored at −20 °C. Samples from these aliquots were used for each assay, to a final 10–50 μM concentration, with no more than 1% (v/v) DMSO in order to keep its impact to insignificant levels.^53^ Conjugate samples were again sonicated twice for 3 min prior to use.

To avoid porphyrin photoactivation and production of reactive oxygen species, all assays described below were performed under no-light conditions: samples were handled on amber containers, with flow chamber lights and, when possible, room lights turned off. Controls under light and no-light conditions were performed to ensure reproducibility.

### Cells and Cell Culture Reagents

bEnd.3 murine brain endothelioma, human embryonic kidney 293T (HEK293T), and TZM-bl cell lines were purchased from ATCC (Manassas, VA, USA). HIV-1 laboratory-adapted strain NL4-3 (HIV-1NL4-3) molecular clone (pNL4-3) was provided by the NIH AIDS Research and Reference Reagent Program, Division of AIDS, NIAID, NIH (Bethesda, MD, USA). Dulbecco’s Modified Eagle’s Medium (DMEM), fetal bovine serum (FBS), penicillin–streptomycin (Pen-Strep), and trypsin-EDTA were obtained from Gibco (Thermo-Fisher, MA, USA). The Luc-Screen luciferase detection system was obtained from Applied Biosystems (Thermo-Fisher, MA, USA). AlamarBlue reagent was purchased from Invitrogen (Thermo-Fisher).

### In Vitro BBB Translocation Assay

An in vitro BBB model consisting of endothelial cells growing on the apical side of a porous membrane was used.^46^ bEnd.3 cells (ATTCC-CRL2299) were cultured as a monolayer on T-flasks in DMEM supplemented with 10% fetal bovine serum (FBS), 1% penicillin/streptomycin. Cells were grown in a humidified atmosphere of 5% CO_2_ at 37 °C (MCO-18AIC (UV), Sanyo, Japan) with the medium changed every other day. Cells were allowed to grow until confluence in a culture T-flask, and then carefully harvested with trypsin-EDTA and seeded (3500 cells/well) onto fibronectin-coated tissue culture 24-well inserts (transparent polyester membrane with 1.0 μm pores) (BD Falcon, USA). The medium was changed every other day for 10 days, after which cells were washed twice with 1× PBS and once with DMEM medium without phenol red. Next, conjugates diluted in DMEM without phenol red to a final concentration of 10 μM were added to the apical side of the in vitro BBB model (Figure 4A). Experiments were performed on different days using independently grown cell cultures.

The translocation of conjugates was determined by fluorescence intensity (λ_exc_ = 380 nm and λ_em_ = 625 nm for MP conjugates, and λ_exc_ = 380 nm and λ_em_ = 675 nm for PP conjugates). P3-CF and P5-CF, peptides labeled with 5(6)-Carboxyfluorescein (CF), were used as positive translocation controls (λ_exc_ = 492 nm and λ_em_ = 517 nm). After 24 h incubation, samples from the apical and basolateral side were collected and analyzed. Fluorescence was measured using a Varioskan Lux plate reader (Thermo Scientific, MA, USA). The percentage (%) of translocation was calculated using the following equation:
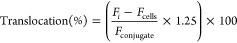
1where *F*_*i*_ is the recovered fluorescence intensity, *F*_cells_ is the recovered fluorescence intensity from cells without the conjugate, and *F*_conjugate_ is the fluorescence intensity of total peptide initially added to the transwell apical side.

### In Vitro BBB Integrity Assay

After 24 h incubation with conjugates, an in vitro integrity assay was performed.^19^ Briefly, cells were washed twice with PBS and once with DMEM medium without phenol red. Then, previously diluted 4 kDa fluorescein isothiocyanate-dextran (FD4) (Sigma-Aldrich, Spain) in DMEM without phenol red to an absorbance of 0.1 was added to the apical side and incubated for 2 h. Samples were collected from the apical and basolateral side, and fluorescence intensity was measured with λ_ext_ = 493 nm and λ_em_ = 520 nm, using a Varioskan Lux plate reader. The integrity of the barrier was determined as follows:
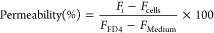
2where *F*_i_ is the recovered fluorescence intensity, *F*_cells_ is the recovered fluorescence intensity from cells without FD4 incubation, *F*_FD4_ is the fluorescence intensity of total FD4 initially added to the transwell apical side, and *F*_Medium_ is the fluorescence intensity of DMEM medium without phenol red.

### Virus Culture

HEK293T and TZM-bl cell lines were cultured in DMEM supplemented with 10% (v/v) FBS and 100 U/mL Pen-Strep (complete medium), and incubated at 37 °C, in a 5% CO_2_ atmosphere. These conditions applied to all cell culture incubation periods.

Recombinant HIV-1_NL4–3_ viruses were produced in HEK293T cell cultures transfected with the pNL4-3 infectious clone through the calcium phosphate coprecipitation method.^54,55^ HEK293T cells were seeded at 5 × 10^5^ cells/well in tissue culture-treated 6-well microplates from TPP (Trasadingen, Switzerland), and incubated for 24 h. To prepare calcium-phosphate-DNA transfection mixtures, pNL4-3 DNA (3.5 μg/well) was initially diluted in 1 mM Tris-HCl, 0.1 mM EDTA, 250 mM CaCl_2_, pH 7.6, and then added, dropwise, to an equal volume of 50 mM HEPES, 280 mM NaCl, 1.5 mM Na_2_HPO_4_, pH 7.05, under gentle agitation. Transfection mixtures were incubated at rt for 20 min before addition to cells. After 18 h, transfection mixtures were replaced with fresh complete medium. Viral supernatants were collected 48 h post-transfection, centrifuged at 315 *g* for 5 min to remove cell debris, and stored at −80 °C until use.

Viral supernatants harvested from pNL4-3-transfected HEK293T cells were titered through the Reed-Muench method^56^ based on HIV-1_NL4–3_ infectivity against TZM-bl cell cultures. TZM-bl cells were seeded at 2 × 10^4^ cells/well in tissue culture-treated 96-well microplates from Corning (Corning, NY, USA), and incubated for 24 h. Cells were then incubated with 2-fold serial dilutions of viral supernatants for 3 h, after which the supernatant was replaced with fresh complete medium. After 45 h, TZM-bl cell infection was quantified through luciferase reporter-gene expression levels, under the control of an HIV-1 TAT-dependent LTR promoter^57^ using the Luc-Screen luciferase chemiluminescence detection system. Luminescence was measured in an Infinite M200 microplate reader from Tecan (Männedorf, Switzerland). Cells were considered to be infected if the respective luminescence intensity (*L*) was 5-fold higher than that of control cells unexposed to virus. Titration was performed with at least four replicates to allow accurate estimation of TCID_50_ in viral supernatants.

### In Vitro Inhibition of HIV-1_NL4–3_ Infection

The inhibitory activity of conjugates on HIV-1_NL4–3_ entry into TZM-bl cells was evaluated as previously described.^58^ TZM-bl cells were seeded at 2 × 10^4^ cells/well in tissue culture-treated 96-well microplates and incubated for 24 h. HIV-1_NL4–3_ viral supernatants (100 or 10 TCID_50_/well) were then incubated for 1 h with 2-fold serial dilutions of either nonconjugated peptide and porphyrins or conjugates, after which the infection mixture was added to TZM-bl cells. Untreated cells (in the absence of peptide–porphyrin conjugate) were used as control. Virions were incubated with cells for 3 h, after which cells were washed to remove unbound virions and new medium was added. After 45 h, infection was quantified by the Luc-Screen chemiluminescence assay. Measurements were performed in an Infinite M200 microplate reader. *L* values were analyzed through nonlinear regression with the classical dose–response relationship (median-effects model based on mass action):^59^
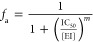
3
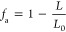
4where *L*_0_ is the luminescence intensity in the absence of the inhibitor, IC_50_ is the concentration that inhibits 50% of viral infection, *m* is a slope parameter equivalent to the Hill slope, and [EI] is the inhibitor concentration. At least three independent experiments were performed for each assay.

### Cell Viability Assay

bEnd.3 cells were plated in fibronectin precoated 96-well flat-bottom clear black polystyrene plate (Corning, New York, USA) as previously described. Cells were then cultured in complete medium at 37 °C in a 5% CO_2_ atmosphere, with medium replaced every 2 days. After 10–11 days, cells were treated with conjugate concentrations in the 6.25–50 μM range for 24 h at 37 °C in a 5% CO_2_ atmosphere. Viability was evaluated by the CellTiter-Blue assay (Promega, Wisconsin, USA), based on resazurin reduction into highly fluorescent resorufin by metabolically active cells. By distinguishing metabolic from nonmetabolic cells, cytotoxicity can be indirectly determined. After incubation, cells were washed with PBS, pH 7.4, and 15 μL of CellTiterBlue reagent in 100 μL of complete medium was added to the cells and incubated for 1.5 h at 37 °C in 5% CO_2_. Fluorescence was measured at 590 nm, with excitation at 560 nm, using an Infinite F200 plate reader (Tecan, Switzerland). Complete medium and medium containing 0.25% Triton X-100 were used as positive and negative controls (100% and 0% viability), respectively. Cell viability (%) was determined using the following equation:
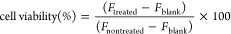
5where *F*_treated_ is the fluorescence emission of conjugate-treated cells, *F*_nontreated_ is the fluorescence emission of the control nontreated cells, and *F*_blank_ is the fluorescent emission of CellTiter reagent in complete medium without cells.

Results are representative of at least three different independent experiments.

### Hemolysis Assay

Human red blood cells (hRBCs) were obtained from human blood samples collected in EDTA tubes on the day of the experiments. 1 mL blood was centrifuged for 5 min at 4000 rpm, 4 °C, and the resulting pellet was washed (3× or until supernatant was clear) with TN buffer (Tris 10 mM, NaCl 150 mM, pH 7.4) by centrifugation at 4000 rpm, 4 °C. The supernatant was discarded, and the hRBC pellets were resuspended in TN buffer (100 μL hRBCs in 20 mL buffer) to give a 0.5% (v/v) hRBC solution. hRBCs were then added to microtiter plate wells containing 2-fold dilutions of conjugate (0.1–50 μM range) in TN buffer (+ 1% DMSO), with a final hRBC concentration of 0.25% (v/v). Plates were incubated for 1 h at 37 °C with 100 rpm stirring, then centrifuged for 5 min at 4000 rpm, 4 °C, and supernatants were transferred to a 96-well flat-bottom polystyrene plate (Corning). Hemolysis was assessed by hemoglobin (Hb) release, measured by absorbance at 415 nm in a Varioskan LUXTM microplate reader (Thermo Fisher Scientific, Inc.). As porphyrin and Hb spectra are partially overlapping, to exclude porphyrin interference in Hb quantification a blank reading of each PPC (no hRBCs, TN buffer +1% DMSO) was subtracted from each measurement. TN buffer (+1% DMSO, no conjugate) and hRBCs with 1% Triton X-100 were used as negative and positive controls, respectively. Hemolysis (%) was determined using the following equation:
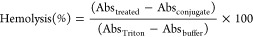
6where Abs_treated_ is the absorbance of hRBC treated with the conjugates, Abs_conjugate_ is the absorbance from each conjugate concentration without hRBCs, Abs_Triton_ is the absorbance of Triton X-100 treated hRBCs, and Abs_buffer_ is the absorbance of TN buffer alone. HC_50_ values were determined by GraphPad Prism 8 software using a log(inhibitor) vs normalized response (variable slope). Experiments were performed using 3 different blood donors.

## Supporting Information Available

Schematic representation of conjugation through Lys side-chain NH_2_ group; list of all synthesized PPCs with their corresponding mass, yield, and purity; TIC-MS raw spectra of all 14 PPCs; HPLC chromatograms at various wavelengths of several PPCs; NMR characterization of P2-MP; MS characterization of some side-reaction products formed during conjugation reaction; conjugation reaction of MP with(out) DIPEA, MP-P3, MP-P5, P4-MP, MP-L-P3, and P4-L-MP cytotoxic effects against TZM-bl cells; PPC internalization into bEnd.3 cells (PDF)
