# Supplementary material for: Penetrating the Blood-Brain Barrier with New Peptide–Porphyrin Conjugates Having anti-HIV Activity
Source: Bioconjug Chem. 2021 May 25;32(6):1067–77. doi: 10.1021/acs.bioconjchem.1c00123 (PMC8485325; doi:10.1021/acs.bioconjchem.1c00123)
Supplement: Supplementary file 1 — bc1c00123_si_001.pdf [file bc1c00123_si_001.pdf]

Penetrating the blood-brain barrier with new peptide-porphyrin conjugates having anti-HIV activity

Diogo A. Mendonça[‡], Mariët Bakker[£], Christine Cruz-Oliveira[‡], Vera Neves[‡], Maria Angeles Jiménez[§], Sira Defaus[†], Marco Cavaco[‡], Ana Salomé Veiga[‡], Iris Cadima-Couto[‡], Miguel A.R.B. Castanho[‡*], David Andreu[†*], Toni Todorovski[†*]

[†]Department of Experimental and Health Sciences, Pompeu Fabra University, Barcelona, Spain; [‡]Instituto de Medicina Molecular, Faculdade de Medicina, Universidade de Lisboa, Lisbon, Portugal; [§]Department of Biological Physical Chemistry, Institute of Physical Chemistry Rocasolano (IQFR-CSIC), Madrid, Spain; [£]Avans University of Applied Sciences, Breda, Netherlands

[*]corresponding author

Supporting Information

CONTENT

Reaction scheme (Scheme S1)………………………………………………………………………… 2

Synthesized conjugates (Table S1) and corresponding TIC-MS spectra……………………………………………………………………………………………3

LC analysis of the putative diastereomeric conjugates……………………………………….9

2D ^{1}H,^{1}H-TOCSY spectra and 2D ^{1}H,^{13}C-HSQC spectra for peptide P2-MP (Table S2, Figures S2 and S3)..11

Mass spectrometry characterization of some side-products formed during various conjugation strategies (Table S3)………………………………………………..14

MPIX coupling reaction with(out) addition of DIPEA………………………………………….15

TZM-bl Cell Viability Studies…………………………………………………………………………….16

PPCs internalization into bEnd.3 cells………………………………………………………………… 18

REFERENCES……………………………………………………………………………………………………. 19

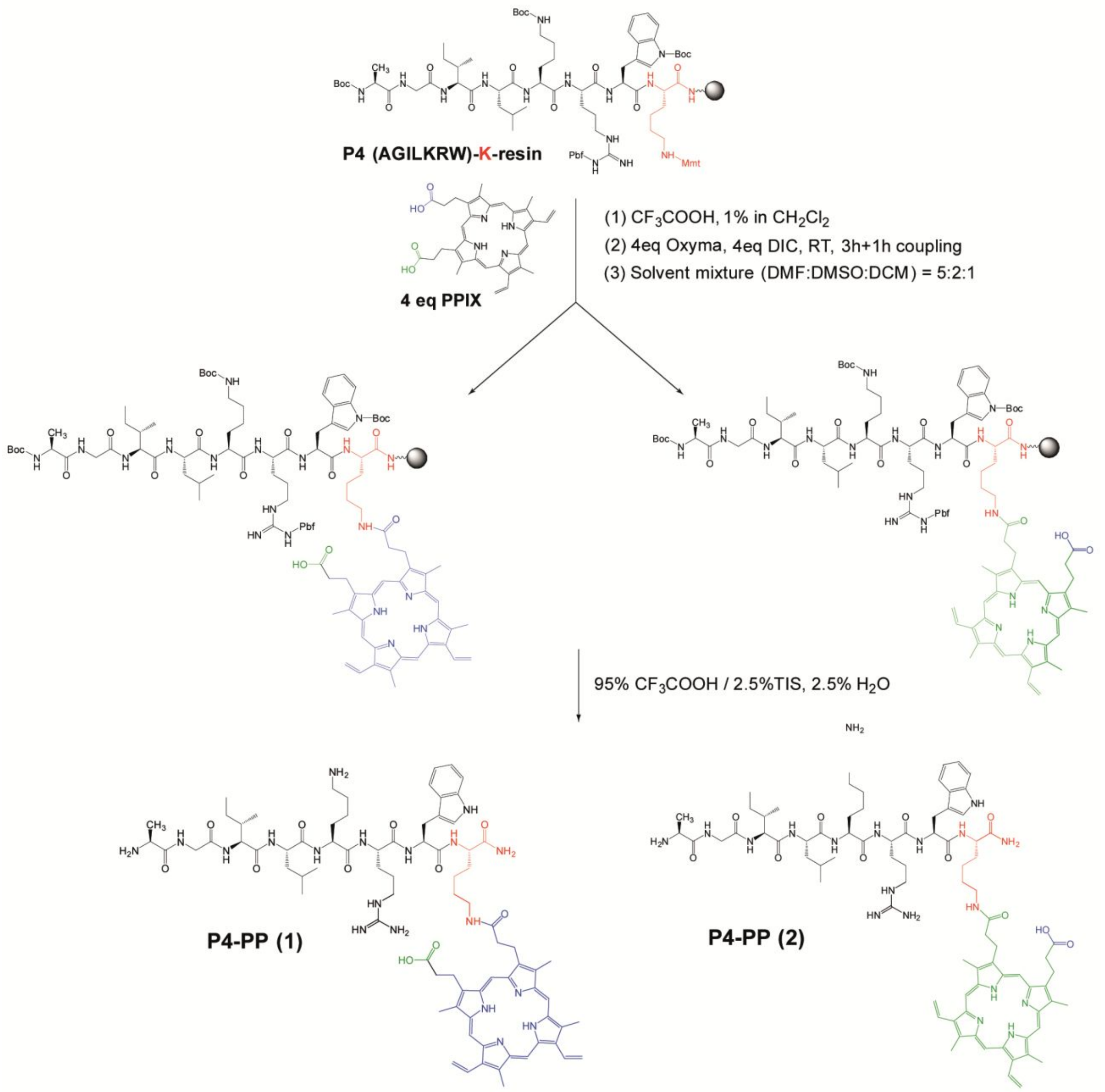

Scheme 1. Schematic representation of on-resin synthesis of P4-PP. The formation of two putative diastereomers is showed through conjugation of -COOH groups of PP with Lys side-chain NH_2 group of P4 peptide.

Table S1. Mass, final yield and HPLC purity of synthesized conjugates.

Conjugate[a]	M+H	M+2H	M+3H	M+4H	Yield[b] (%)	Purity (%)
P2-PP	1892.1	946.6	631.4	473.8	14.2	92.4
P2-MP	1896.1	948.6	632.7	474.8	15.1	98.7
PP-P1	1762.9	881.9	588.3	441.5	14.8	93.8
MP-P1	1767	884	589.7	442.5	13	97.5
P4-PP	1515.9	758.4	505.9	379.7	13.6	91.7
P4-MP	1519.9	760.5	507.3	380.7	16.6	92.7
PP-P3	1386.8	693.9	462.9	347.5	19.5	93.4
MP-P3	1390.8	695.9	464.3	348.5	14.3	91.8
MP-L-P3	1536.9	768.9	512.9	384.9	20.8	95.7
P4-L-MP	1664.9	832.9	555.6	416.9	15.6	94.6
PP-L-P3	1532.8	766.9	511.6	383.9	24.8	94.7
P4-L-PP	1660.9	830.9	554.3	415.9	15.9	96.3
MP-P5	1360.6	680.8	454.2	340.9	28.8	94.8
P6-MP	1488.7	744.9	496.9	372.9	27.7	97.6

[a]: L stands for an O_2Oc spacer residue

[b]: Prepared by the optimized protocol (DIC/oxyma 3h+1h)

P2-PP

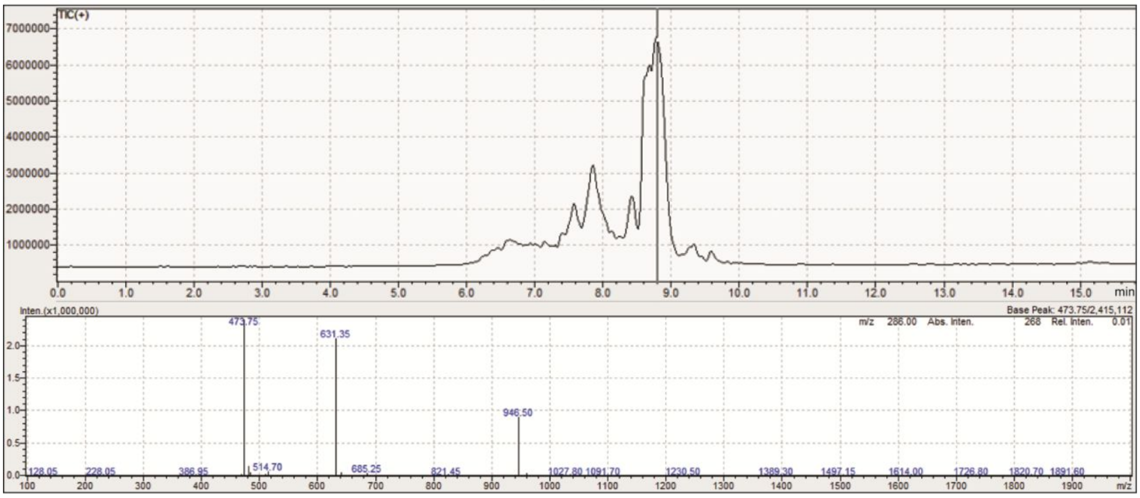

P2-MP

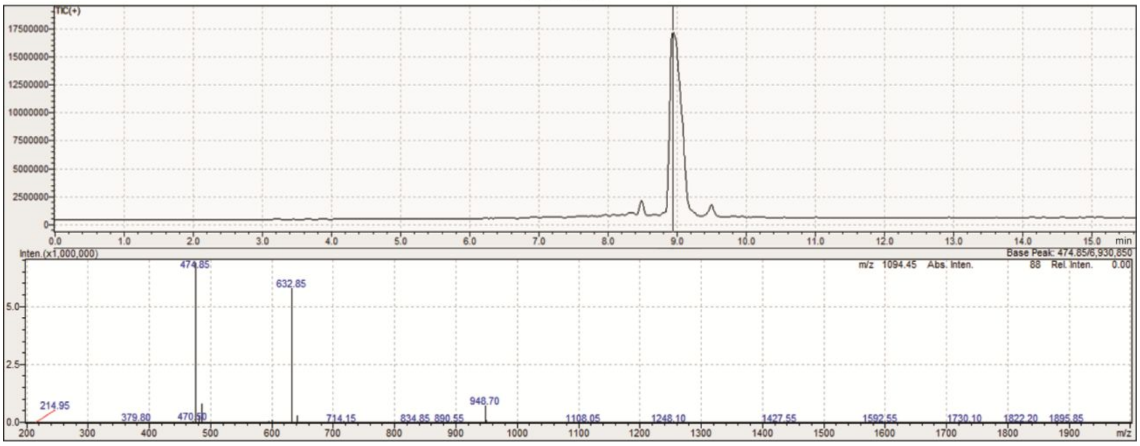

PP-P1

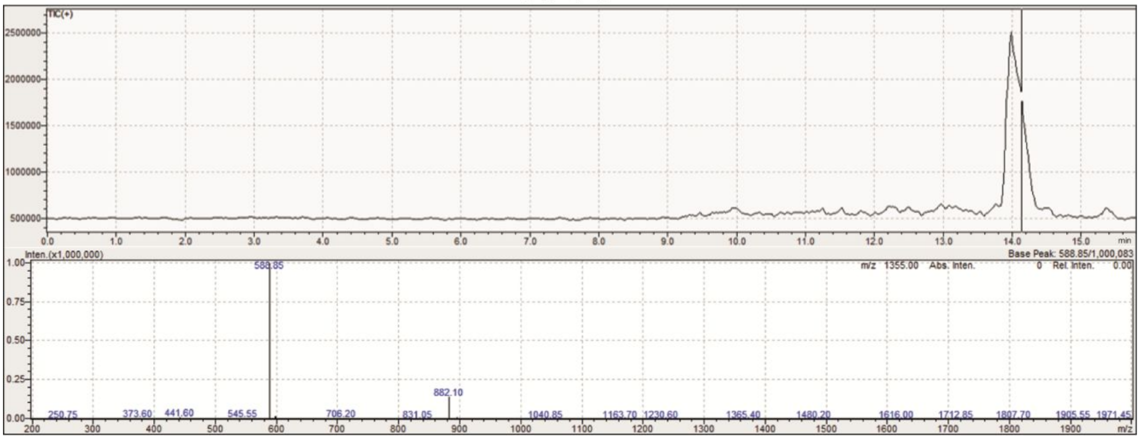

MP-P1

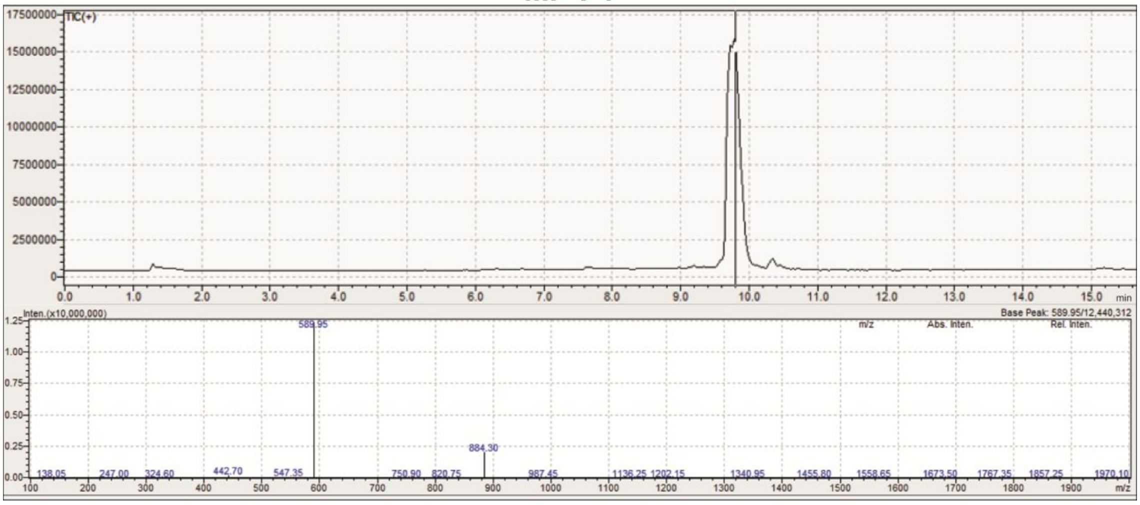

P4-PP

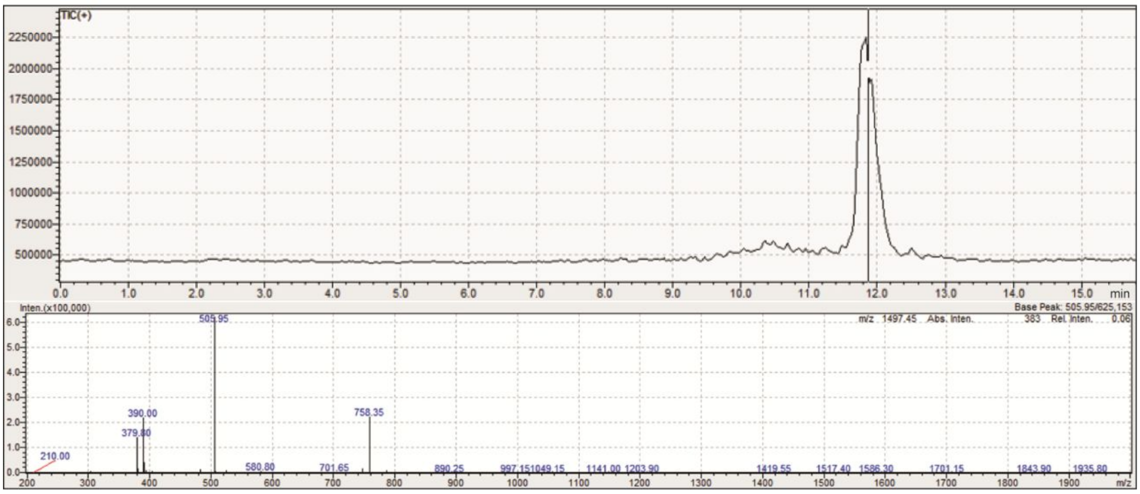

P4-MP

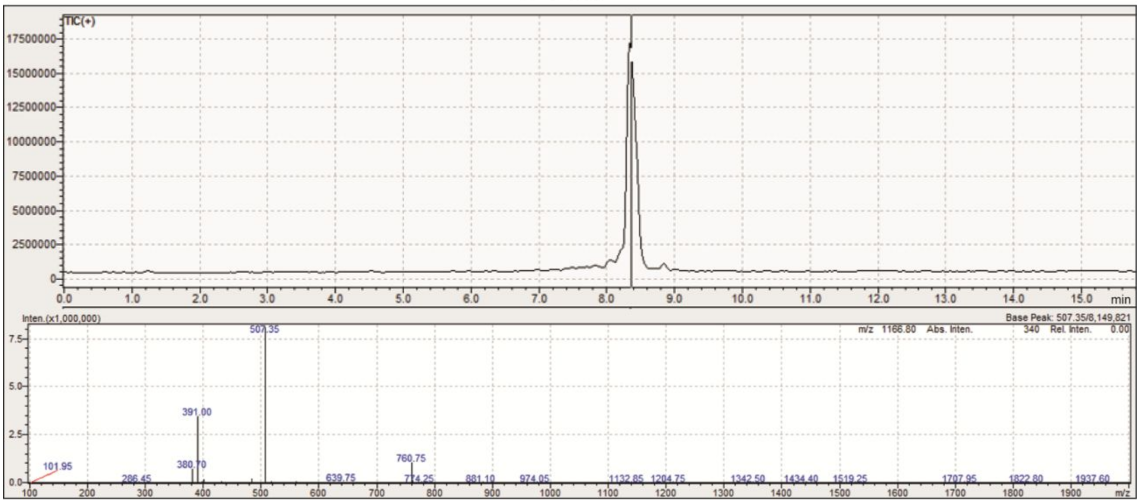

PP-P3

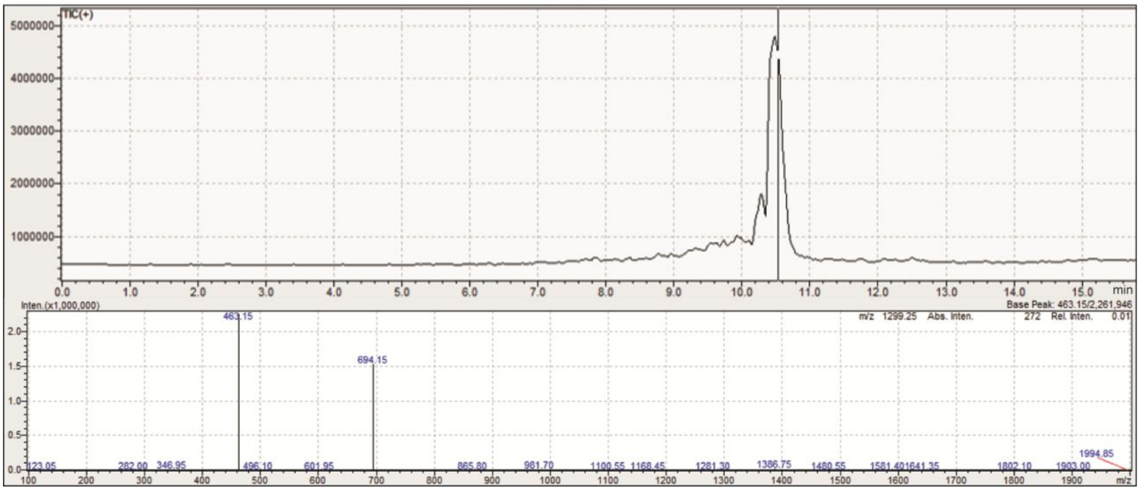

MP-P3

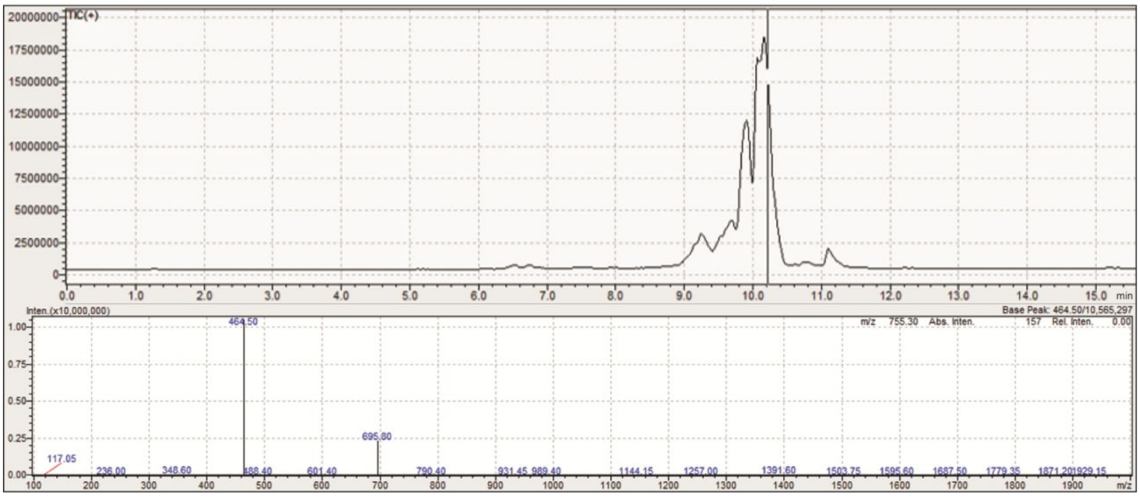

MP-L-P3

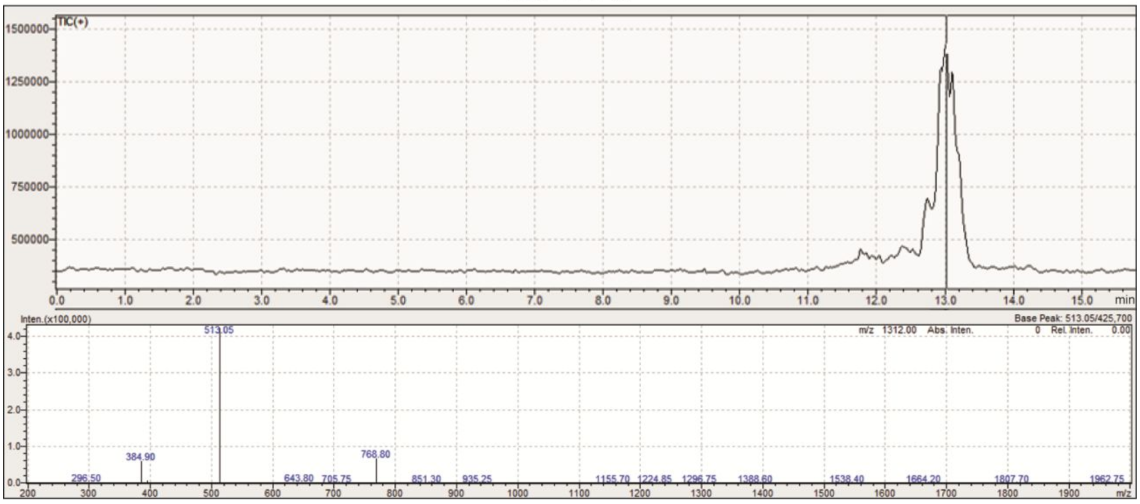

P4-L-MP

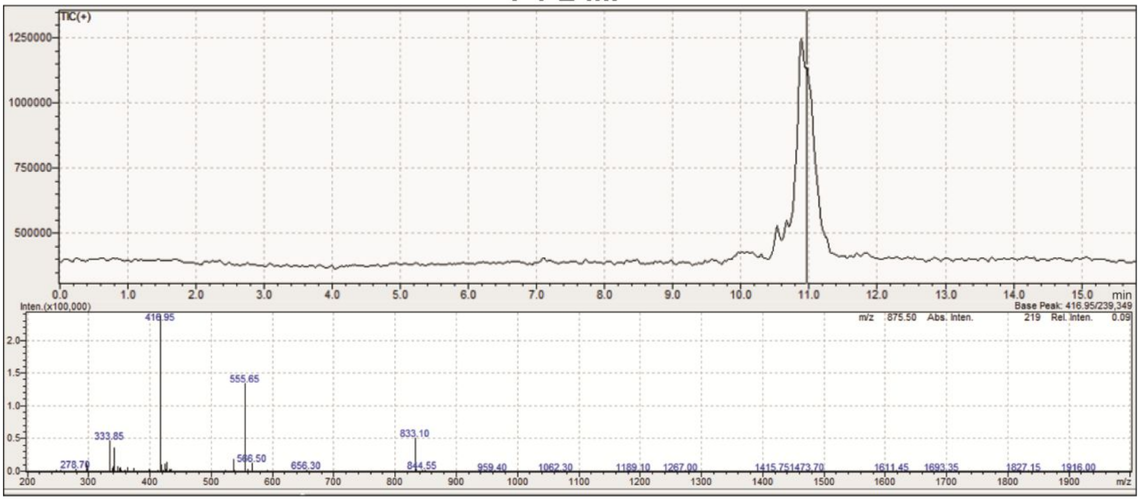

PP-L-P3

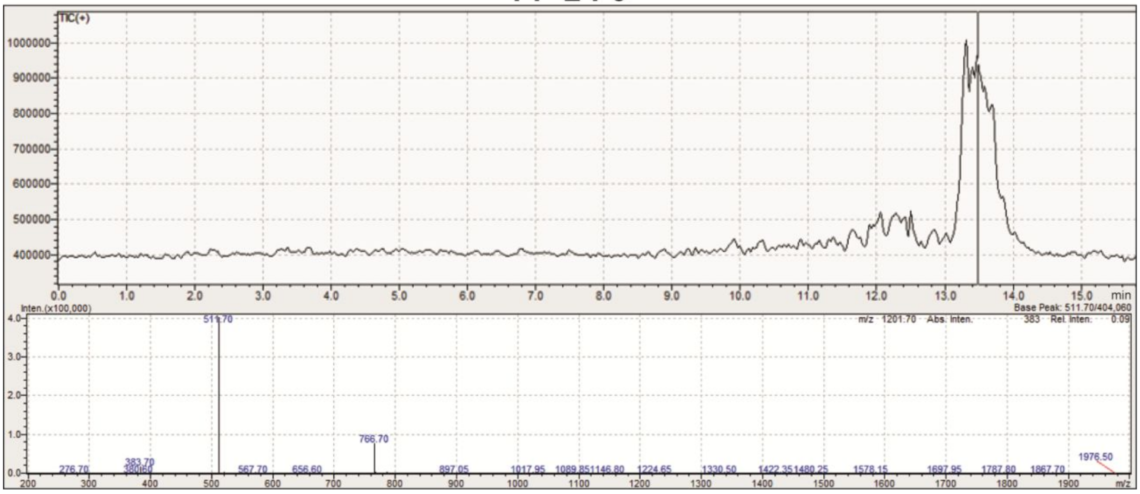

P4-L-PP

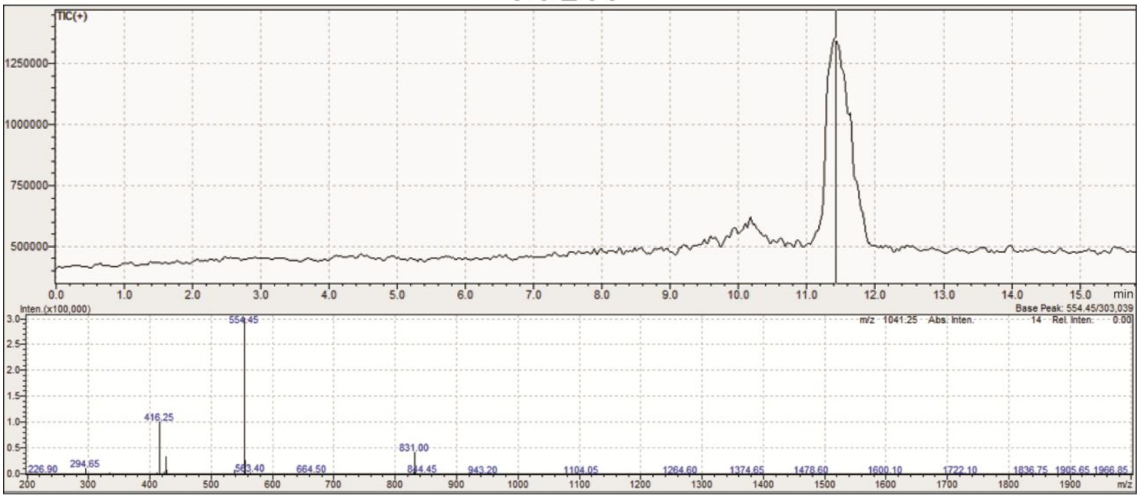

MP-P5

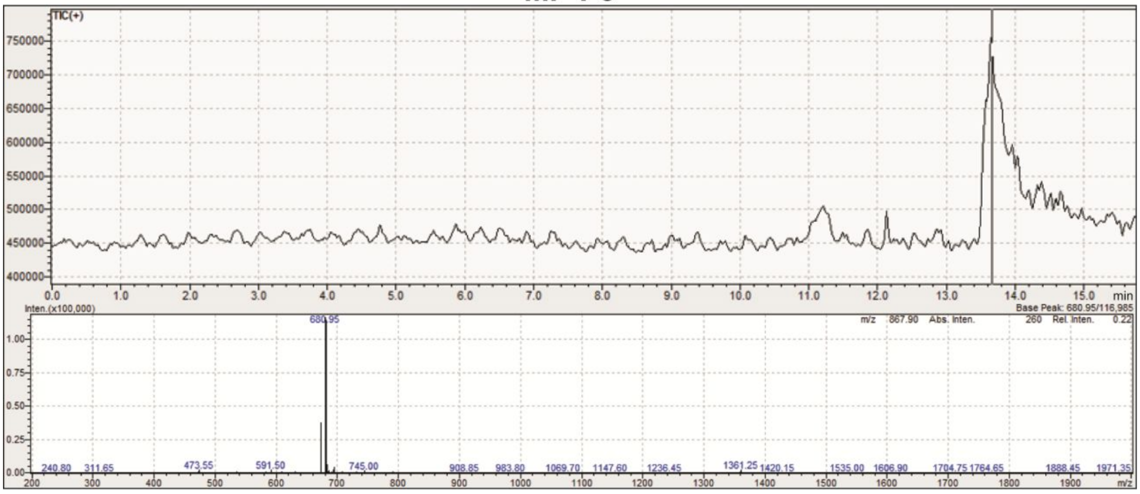

P6-MP

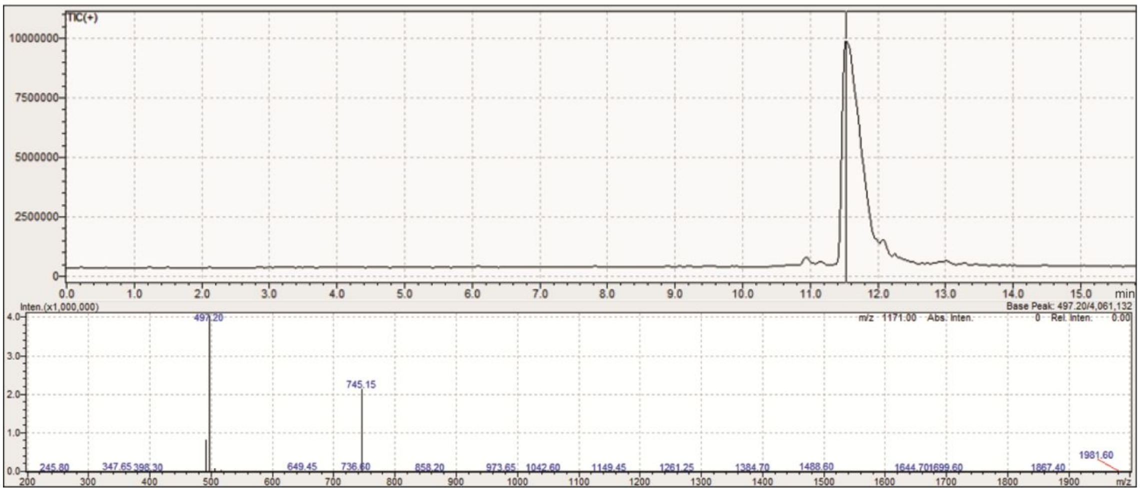

Figure S1. Raw TIC-MS spectra of all 14 synthesized PPCs. The order of presented spectra is identical with the order of the conjugate in Table S1.

LC ANALYSIS

The formation of the putative diastereomeric species (see scheme S1) was evaluated though LC analysis using various ion-pair reagents and detection wavelengths. The conjugates (P2-MP, P2-PP and P4-MP) were dissolved in 25% MeCN/H_2O at final concentration of 1 mg/mL. Each conjugate solution (5 µL injected) was analyzed by a linear 25%-70% MeCN (into 0.1% TFA, or 0.1% HFBA, in H_2O) gradient over 45 min using PDA detection. In all tested cases, only one elution peak corresponding to the synthesized conjugate, was observed.

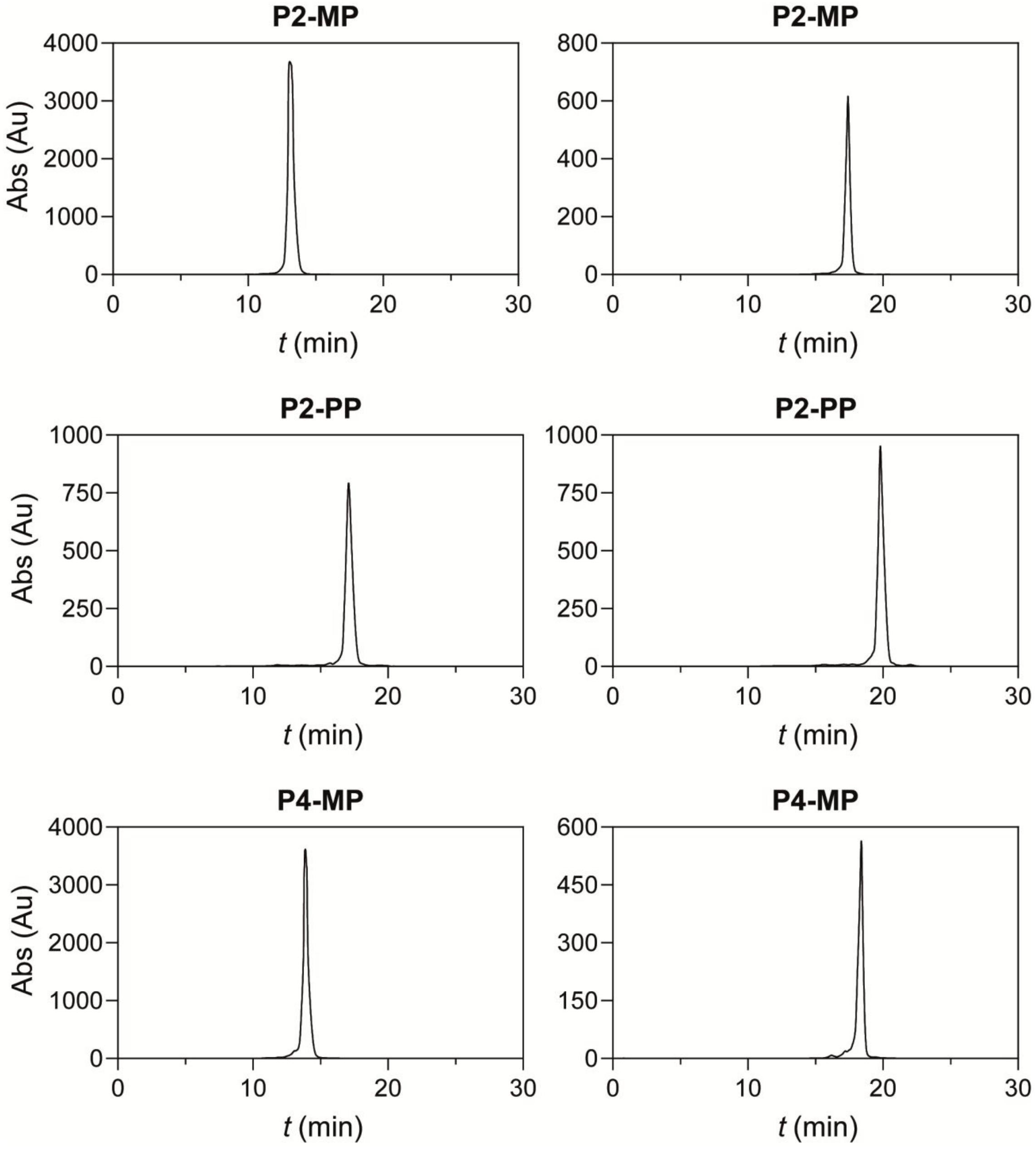

Figure S2. LC chromatograms of P2-MP, P2-PP and P4-MP using TFA (left chromatograms) or HFBA (right chromatograms) as ion-pair reagent. The detection wavelength, 401 nm, is maximum absorption wavelength of PP and MP.

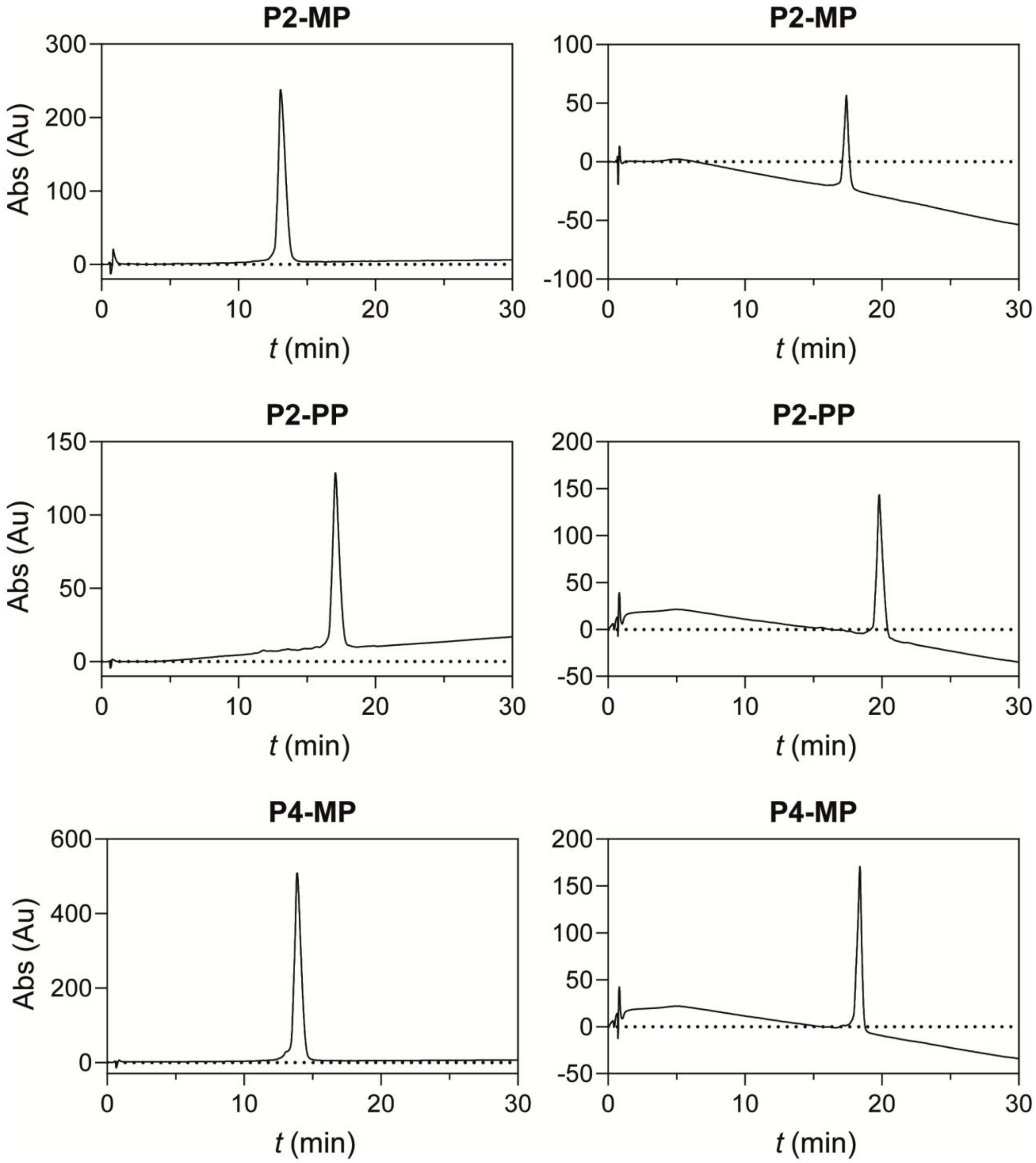

Figure S3. LC chromatograms of P2-MP, P2-PP and P4-MP using TFA (left chromatograms) or HFBA (right chromatograms) as ion-pair reagent. Detection was done at wavelength of 220 nm.

Table S2. ^{1}H and ^{13}C chemical shifts (ppm) for peptide P2-MP in H_2O/D_2O 9:1 at pH 3.1 and 45 °C.

Residue	H^N	$C_\alpha H$	$^{13}C_\alpha$	$C_\beta H$	$^{13}C_\beta$	Other (^{13}C)
V1		3.87	61.4	2.24	32.8	$C_\gamma H_3$ 1.04 (19.7); $C_{\gamma'}H_3$ 1.04 (20.4)
Q2	8.65	4.43		2.01, 2.08	29.8	$C_{\gamma\gamma'}H$ 2.37, 2.37 (33.9); $N_{\varepsilon\varepsilon'}H_2$ 6.82, 7.46
Q3	8.51	4.36		1.97, 2.07	29.6	$C_{\gamma\gamma'}H$ 2.36, 2.36 (33.7); $N_{\varepsilon\varepsilon'}H_2$ 6.78, 7.39
L4	8.32	4.36		1.55, 1.62	42.5	$C_\gamma H$ 1.59 (27.1); $C_\delta H_3$ 0.84 (23.6); $C_{\delta'}H_3$ 0.89 (24.9)
T5	7.94	4.18	62.0	4.10	69.8	$C_\gamma H_3$ 1.12 (21.6)
K6	8.02	4.10	56.3	1.56, 1.56	32.9	$C_{\gamma\gamma'}H$ 1.22, 1.28 (24.7); $C_{\delta\delta'}H$ 1.56, 1.56 (29.0); $C_{\varepsilon\varepsilon'}H$ 2.89, 2.89 (42.2); $N_\zeta H_3$ 7.42
R7	7.81	3.91	56.0	1.36, 1.36	30.6	$C_{\gamma\gamma'}H$ 1.19, 1.19 (26.8); $C_{\delta\delta'}H$ 2.87, 2.87 (43.2); $N_\varepsilon H$ 6.84
F8	nd	4.05	nd	3.06, 3.06	39.7	
F8’	nd	3.89	nd	2.98, 2.98	39.6	
S9	7.33	3.61	57.5	3.11, 3.19	63.3	
S9’	*7.30*	*3.56*	*57.6*	*3.10, 3.13*	*63.2*	
L10	6.82	nd	nd	2.17, 2.37	41.2	$C_\delta H_3$ 0.15 (22.8); $C_{\delta'}H_3$ 0.23 (24.4)
L10’	*nd*	*nd*	*nd*	*nd*	*nd*	*$C_\delta H_3$ 0.12 (22.8); $C_{\delta'}H_3$ 0.19 (24.3)*
K11	7.50	3.84	57.1	1.97, 1.97	nd	$C_{\varepsilon\varepsilon'}H$ 2.87, 2.87 (40.6)
K11’	*7.49*	*3.77*	*57.2*	*1.82, 1.94*	*nd*	

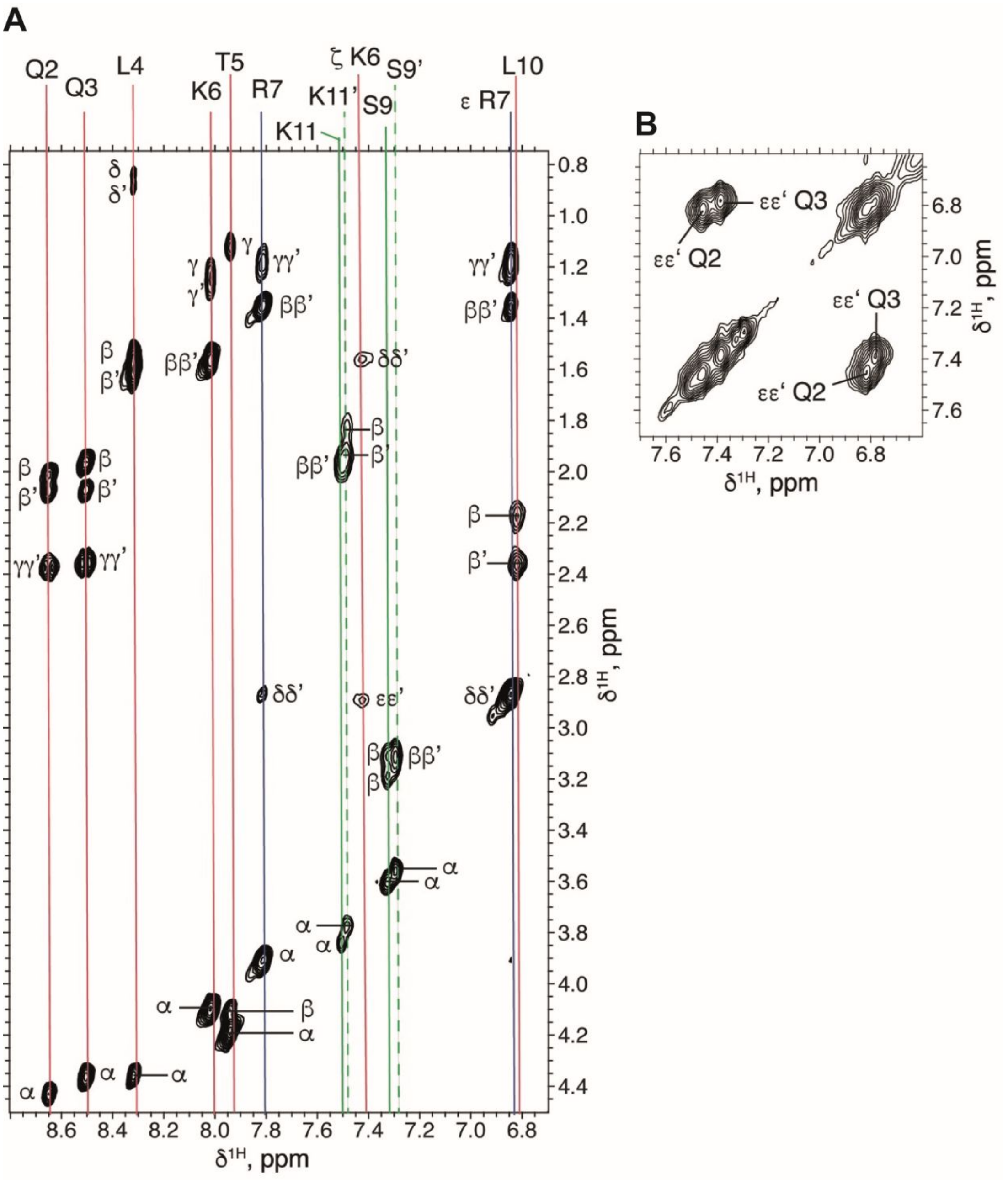

Figure S4. 2D ^{1}H,^{1}H-TOCSY spectra for peptide P2-MP (2 mM) in H_2O/D_2O 9:1 at pH 3.1 and 45 °C. (A) Region showing the cross-peaks are between the H^N amide proton (their ^{1}H chemical shifts can be measured at the horizontal x-axis) and the aliphatic side chain protons, which are labelled by Greek letters (as in Table S2; their ^{1}H chemical shifts can be measured at the vertical y-axis). Cross-peaks corresponding to the same amino acid residue are linked by vertical lines. The cross-peaks belonging to the $N_{\zeta}H_3$ amino group of K6 side chain and to the $N_{\varepsilon}H$ guanidinium proton of the R7 side chains are also labelled. (B) Region showing the cross-peaks for the pair of $N_{\varepsilon\varepsilon'}H_2$ amide protons of the two glutamine residues (Q2 and Q3; see Table S2), which are symmetrical relative to the diagonal.

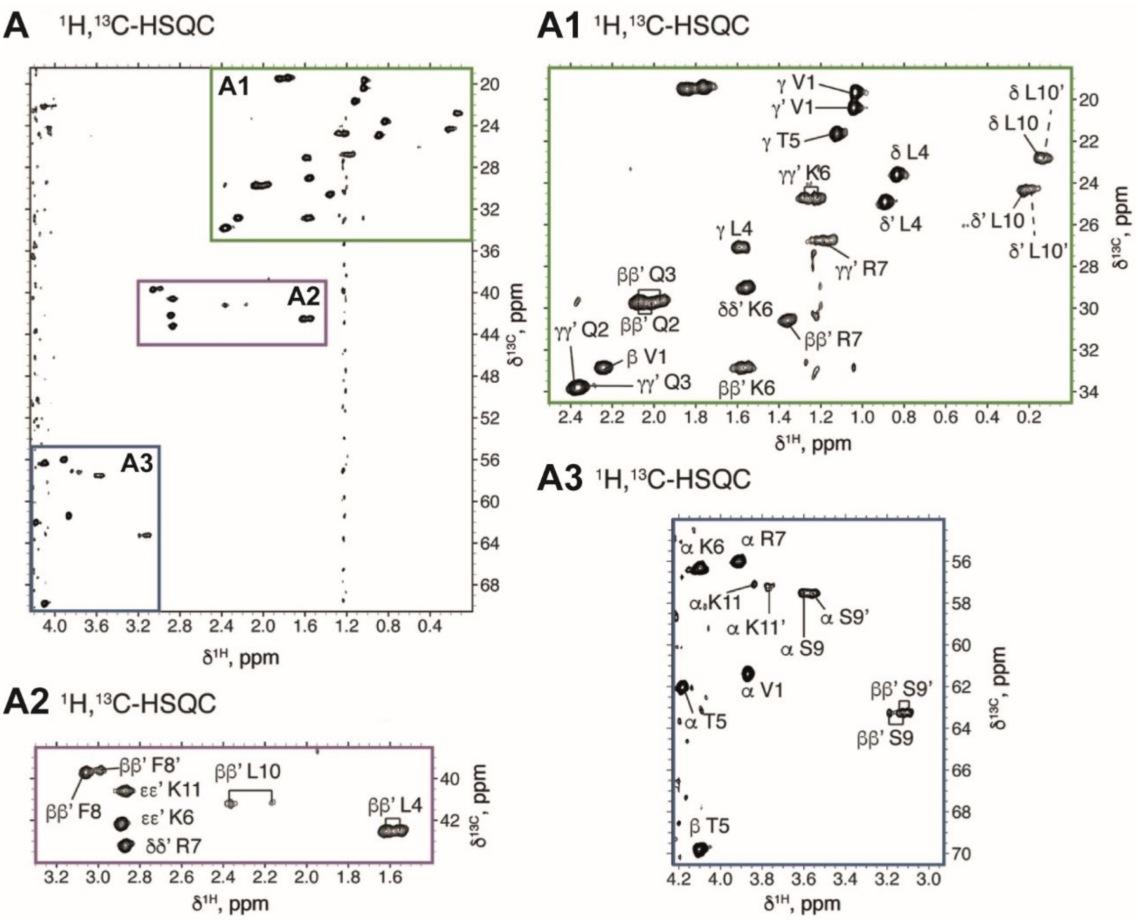

Figure S5. 2D $^{1}H,^{13}C$-HSQC spectra for peptide P2-MP (2 mM) in H_2O/D_2O 9:1 at pH 3.1 and 45 °C. (A) Full aliphatic region; (A1) green rectangle in A; (A2) purple rectangle in A; (A3) blue rectangle in A. Cross-peaks, which correspond to directly bound ^{1}H and ^{13}C nuclei, are labelled indicating the type of proton (Greek letter as in Table S2) and the amino acid they belong to. ^{1}H and ^{13}C chemical shifts can be measured at the horizontal and vertical axis, respectively.

MASS SPECTROMETRY

We were able to assign the masses of some side-reaction products formed during the conjugation reactions with various activation strategies. For example, in PyBOP/DIPEA activation, the peaks at 10.6 min (*m/z* 1444.8, Figure 2a) and 10.5 min (*m/z* 1816.9, Figure 2d), have respectively an *m/z* increase of +54 Da relative to the target conjugate. By performing tandem MS of this product and assigning the a-series of ions (data not shown), we could identify that the modification occurred on the porphyrin section of the molecule (Table S3), though further detailed characterization was not pursued. Similarly, in the case of HATU/DIPEA activation, the side product that co-elute within the conjugate peak was assigned with a mass increase of +27 Da, compared to the mass of the corresponding conjugate. This product was also present in all tested activation protocols, but in much lower extent. Based on the MS/MS spectra the modification of +27 Da can also be related with the porphyrin molecule (Table S3). Moreover, the peak at 8.7 min (*m/z* 2214.8, Figure 2a-c) and peak at 8.6 min (*m/z* 2963.9, Figure 2d-f), correspond to double conjugation i.e conjugate with two peptide molecules per one porphyrin molecule (Table S3, mass increase of +824 Da and +1201 Da). Furthermore, some of the formed side products were related with the peptide part of conjugate. For example, when P3 was used in the conjugation reaction there is appearance of one additional peak with mass increase of +32 Da that elutes immediately before the conjugate peak (*m/z* 1422.8, Figure 2a-c). This peak probably corresponds to oxidation of the Trp residue, that is most susceptible residue for oxidation in the sequence, as the mass difference of +32 Da can be attributed to one of the well-characterized Trp-oxidation products.[1–5] Tandem mass spectra of this oxidized product revealed the full a- and b-series of ions with no detectable peaks corresponding to oxidized product. In contrary, the y-series of ions displayed peaks with +32 Da mass increase confirming that modification occurred at the corresponding Trp residue (data not shown).

Table S3. LC-MS detected side-products using various conjugation chemistries.

	Modification occurred at:				
	Peptide part		Porphyrin part		
Side-product mass increase*(Da)	P1	P3	MP	PP	Conjugation chemistry
+27	X	X	√	√	HATU/DIPEA>PyBoP/DIPEA>DIC/oxyma
+32	X	V	X	X	In all of them
+54	X	X	V	V	PyBoP/DIPEA
+824	X	X	V	V	In all of them
+1201	X	X	V	V	In all of them

*relative to the mass of the conjugate

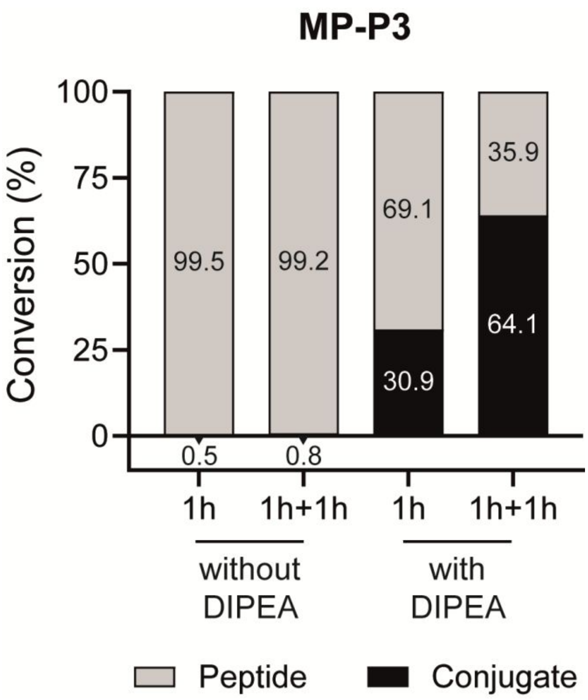

Figure S6. DIPEA relevance on conversion rate with MPIX hydrochloride as porphyrin coupling partner. Bars refer to peptide (grey) and conjugate (black) integrated areas in the HPLC chromatograms of the reaction crudes.

TZM-bl Cell Viability Studies

The cytotoxic effects of each PPC on TZM-bl cell cultures were assessed to ensure that HIV inhibition was a result of the compounds effect on the virus and not on the host cells used in the assay. A resazurin reduction fluorometric assay was performed. Resazurin, the active compound in alamarBlue®, is a blue dye that can be reduced to a pink fluorescent intermediate, resorufin, as a result of cell metabolic activity.[6] TZM-bl cells were seeded at $2x10^4$ cells/well in tissue culture-treated 96-well microplates and incubated for 24 h. Cells were then incubated with serial dilutions (2-fold) of single peptide and porphyrins, or PPC, for 3 h, after which the mixture was replaced with fresh complete medium. Untreated cells (in the absence of peptide-porphyrin combinations) were used as a control. After 42 h, media was replaced with alamarBlue® reagent and incubated for an additional 3 h. Resazurin reduction was quantified by resorufin fluorescent emission intensity, measured in an Infinite M200 microplate reader. Fluorescence emission intensity was collected at 590 nm, using a fixed λ_{exc} of 560 nm. Excitation and emission slits were 9 and 20 nm, respectively. Cell viability was calculated through the following formalism:

$$\% \text{ of viable cells} = \frac{I_{EI} - I_{background}}{I_{control} - I_{background}} \times 100\% \qquad (1)$$

where I_{EI} corresponds to the resorufin fluorescence emission intensity in the presence of EIs or combination, $I_{control}$ to the fluorescence emission intensity in the absence of EIs and $I_{background}$ to the background fluorescence emission from the non-reduced alamarBlue® reagent.

For clarity reasons, only the PPCs that displayed significant HIV inhibition: MP-P3, MP-P5, P4-MP, MP-L-P3 and P4-L-M - are presented here. PPCs did not alter significantly TZM-bl cell viability on the concentration range tested.

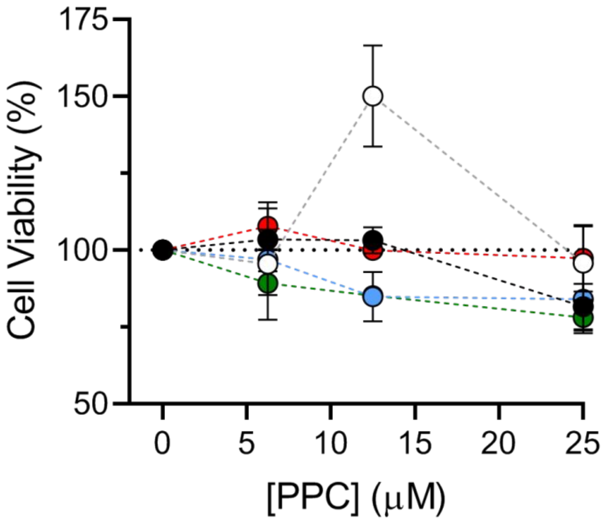

Figure S7. PPCs cytotoxic effects against TZM-bl cells. A resazurin reduction fluorometric assay, dependent on the cell metabolic activity, was used. Cell viability, normalized to the control in the absence of PPC, was measured against three concentrations of each individual PPC: MP-P3 (black), MP-P5 (white), P4-MP (red), MP-L-P3 (blue) and P4-L-MP (green).

PPCs internalization into bEnd.3 cells

bEnd.3 cells were seeded 50000 cells/500 µL in 24-well flat-bottomed plates (Corning, New York, USA) for 24h. Then, medium was removed and cells were washed twice with PBS and once with medium, and incubated for 24 h with different PPCs, at a final concentration of 10 µM in a humidified atmosphere of 5% CO_2 at 37 °C. Finally, cells were collected and washed twice with PBS. The mean fluorescence intensity of 10000 cells was analyzed with BD LSRFortessa X-20 flow cytometer (BD Biosciences, San José, USA) - Laser Violet (405 nm) and detector BV650 (670/30 nm). All experiments were performed in duplicates in three independent days.

In order to elucidate the internalization potential of the PPC, an internalization assay was performed. bEnd.3 cells incubated with 10 µM PPC or porphyrins for 24 h, and internalization was assessed using flow cytometry. Assuming that the fluorescence quantum yield of the PPC is similar to that of porphyrins, we used the cells mean fluorescence intensity (MFI) to quantity PPC internalization. The results (Figure S8) show that the peptide conjugation enhances porphyrin delivery into cells.

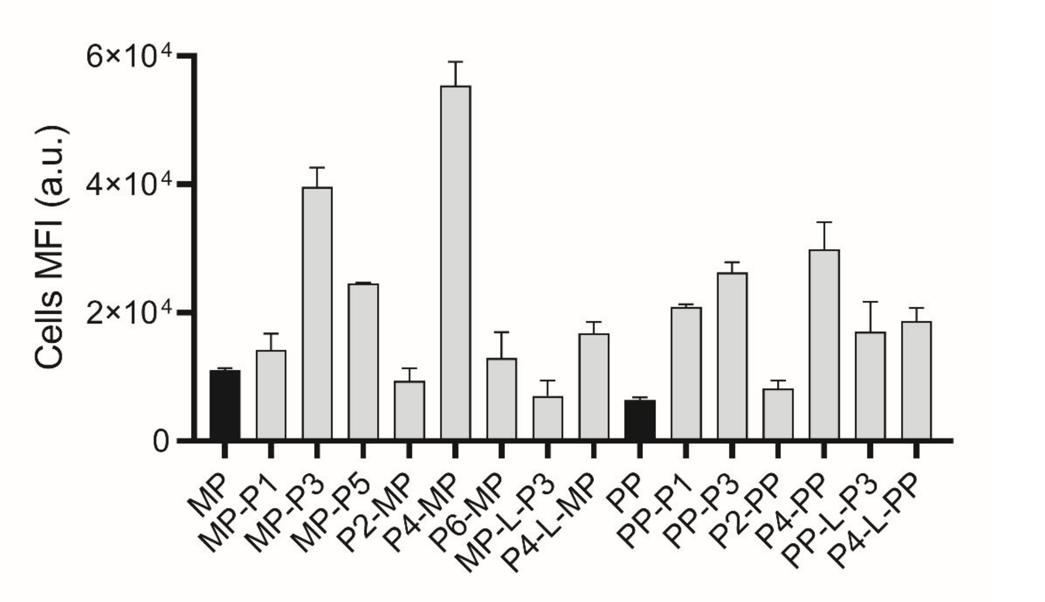

Figure S8 - PPCs internalization in bEnd.3 cells. The cells were incubated with 10 µM PPC or porphyrins for 24 h, and internalization was assessed using flow cytometry. Cells mean fluorescence intensity (MFI) was used to quantify PPCs internalization.

REFERENCES

(1) Bonifay, V., Barrett, T. J., Pattison, D. I., Davies, M. J., Hawkins, C. L., and Ashby, M. T. (2014) Tryptophan oxidation in proteins exposed to thiocyanate-derived oxidants. *Arch. Biochem. and Biophys. 564,* 1-11.

(2) Hinterholzer, A., Stanojlovic, V., Regl, C., Huber, C. G., Cabrele, C., and Schubert, M. (2020) Identification and quantification of oxidation products in full-length biotherapeutic antibodies by NMR spectroscopy. *Anal. Chem. 92,* 9666 – 9673.

(3) Triquigneaux, M. M., Ehrenshaft, M., Roth, E., Silman, I., Ashani, Y., Mason, R. P., Weiner, L., and Deterding, L. J. (2012) Targeted oxidation of torpedo californica acetylcholinesterase by singlet oxygen: Identification of N-formylkynurenine tryptophan derivatives within the active-site gorge of its complex with the photosensitizer methylene blue. *Biochem. 448,* 83 – 91.

(4) Todorovski, T., Fedorova, M., Hennig, L., and Hoffmann, R. (2011) Synthesis of peptides containing 5-hydroxytryptophan, oxindolylalanine, *N* -formylkynurenine and kynurenine. *J. Pept. Sci. 17,*256 – 262.

(5) Todorovski, T., Fedorova, M., and Hoffmann, R. (2011) Mass spectrometric characterization of peptides containing different oxidized tryptophan residues. *J. Mass Spectrom. 46,* 1030 – 1038.

(6) O'Brien, J., Wilson, I., Orton, T., and Pognan, F. (2000) Investigation of the alamar blue (resazurin) fluorescent dye for the assessment of mammalian cell cytotoxicity. *Eur. J. Biochem. 267,* 5421 – 5426.
